# Upcycling of Chitin to Cross‐Coupling Catalysts: Tailored Supports and Opportunities in Mechanochemistry

**DOI:** 10.1002/cssc.202401255

**Published:** 2024-10-21

**Authors:** Oscar Trentin, Daniel Ballesteros‐Plata, Enrique Rodríguez‐Castellón, Leonardo Puppulin, Maurizio Selva, Alvise Perosa, Daily Rodríguez‐Padrón

**Affiliations:** ^1^ Department of Molecular Science and Nanosystems Ca' Foscari University of Venice Via Torino 155 30175 Venezia Mestre Italy; ^2^ Department of Inorganic Chemistry Facultad de Ciencias, Instituto Interuniversitario de Biorrefinerías I3B Universidad de Málaga Campus de Teatinos s/n 29071 Málaga Spain

**Keywords:** Chitin, *N*-doped carbon catalysts, Cross-coupling reactions, Mechanochemistry, Structure-performance relationship

## Abstract

In this study chitin derived from shrimp shells was used in the design of heterogeneous Pd‐based catalysts for Heck and Suzuki‐Miyaura cross‐coupling reactions. The synthesis of Pd nanoparticles supported on *N*‐doped carbons was performed through different approaches, including a sustainable mechanochemical approach, by using a twin‐screw extruder. All catalytic systems were characterized by a multitechnique approach and the effect of nanoparticles size, *N*‐doping on the support, and their synergistic interactions were elucidated. Specifically, Kelvin Probe Atomic Force Microscopy provided valuable insights on charge transfer and metal‐support interactions. The catalytic behaviour of the samples was investigated in cross‐coupling reactions under batch conditions and under semi‐continuous flow solvent‐free conditions, respectively obtaining a quantitative yield and a noteworthy productivity of 8.7 mol/(g_Pd_h).

## Graphical abstract

Chitin‐derived palladium‐based catalytic systems: examining the effects of nanoparticle size, N‐doping in the support, and their synergistic interactions in Heck‐Mizoroki and Suzuki‐Miyaura cross‐coupling reactions, using sustainable protocols involving biomass‐derived solvents or solvent‐less mechanochemistry through extrusion.

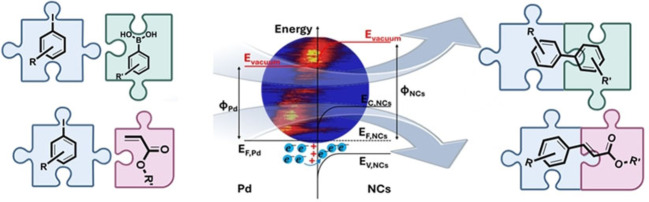

## Introduction

Biomass, a key renewable carbon source, plays a vital role in transitioning to sustainable practices by facilitating the production of chemicals, materials, and biofuels.[^1^, ^2^] In the seafood industry, a significant portion of biomass is passed overlooked, particularly in crustacean processing, where around 50 % of the weight is discarded annually, yielding 6 to 8 million tons of waste from crab, shrimp, and lobster shells.[^3^, ^4^, ^5^] Despite its potential, only about 5 % of this waste is repurposed for animal feed, leaving the majority untreated and disposed of in ways that contribute to environmental pollution and pose health risks. This large amount of waste provides an opportunity for the extraction and utilization of chitin, the second most abundant biopolymer, with an annual production of 100 billion tonnes.[^6^, ^7^, ^8^, ^9^]

Chitin (poly‐beta‐1,4‐N‐acetylglucosamine) contains approximately 6–7 % nitrogen (C : N =8 : 1), rendering it a promising candidate for environmental catalysis,[^10^, ^11^, ^12^, ^13^] in particular as a precursor for metal‐free nitrogen‐doped carbon catalysts.[^14^, ^15^] Carbon‐based materials are generally employed as supports, for heterogeneous catalysts.^[16]^ However, the use of metal nano‐catalysts supported on carbonaceous materials poses challenges, including nanoparticle agglomeration and leaching due to weak metal‐carbon interactions.^[17]^

With the potential of heteroatoms to enhance catalyst stability and performance, this study explores the synthesis of catalytic systems incorporating palladium nanoparticles supported on chitin derived *N*‐doped carbons. Chitin was sourced from shrimp shells, and the aim was to explore the beneficial attributes of *N*‐doped carbonaceous materials as supports in catalysis.

Palladium is widely recognized as the catalyst of choice for cross‐coupling reactions, although alternative metals such as Pt, Cu, Au, Fe, and Ni have also been investigated.^[18]^ Cross‐coupling reactions hold significant importance due to the possibility of forming new C−C bonds enabling the synthesis of increasingly complex organic frameworks.^[19]^ The pharmaceutical sector, in particular, has greatly benefited from these reactions. In 2006, 11 % of the reactions applied in three multinational pharmaceutical companies (AstraZeneca, Pfizer, and GlaxoSmithKline) were cross‐coupling reactions.^[20]^ Examples include the Heck‐Mizoroki, Suzuki‐Miyaura, Sonogashira, Stille, and Buchwald‐Hartwig (hetero‐coupling C−N) reactions. In particular, this study focuses on the design of green approaches for Heck‐Mizoroki and Suzuki‐Miyaura reactions through heterogeneous catalysis, employing low‐loading metallic Pd nanoparticles supported on *N*‐doped carbons derived from chitin.

Heterogeneous catalysts, which can be easily recycled and reused, are already veered at improving the process sustainability. However, in the context of organic synthesis and catalysis, the greening of other aspects, such as synthetic procedures and the solvent usage, must also be considered. In this context, mechanochemistry offers opportunities that span from synthesizing valuable chemicals to designing advanced nanomaterials.[^21^, ^22^, ^23^] For process scale‐up and intensification, continuous‐flow processes with twin‐screw extruders not only they offer better reaction control, but they also minimize environmental impact, thus reducing production costs.[^24^, ^25^] With this concept in mind, we developed a mechanochemistry strategy via extrusion for both catalyst synthesis and for cross‐coupling reactions.

## Results and Discussion

### General

**Figure 1:**
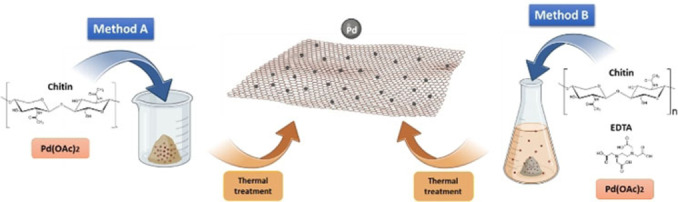
Schematic representation of the synthetic strategies for the preparation of Pd/CN materials: Method A by impregnation, Method B in solution.

Three different strategies were employed to incorporate palladium nanoparticles into the chitin matrix. Chitin extracted from shrimp shells was selected as a sustainable source of nitrogen and carbon. Despite its low solubility, which is often a challenge, the present work proposes a strategy to harness its potential, thereby adding value to a byproduct of the seafood supply chain, in line with the principles of the circular economy. Figure 1 illustrates two of the herein proposed strategies (See Experimental Section), while a separate section will be dedicated to the mechanochemical protocol.

In both protocols in Figure 1, the proper amount of Pd(OAc)_2_ was employed to achieve Pd loadings of 1 %, 2.5 % and 5 % leading to catalytic systems labelled as 1Pd/CNi, 2.5Pd/CNi, 5Pd/CNi for those obtained with method A, and 1Pd/CNs, 2.5Pd/CNs, 5Pd/CNs for those obtained with method B, respectively. Characterization of the catalytic systems was accomplished by a multi‐techniques approach, including XRD, XPS, TEM, SEM and N_2_ physisorption measurements. Details regarding materials characterization are included in the Supporting Information File (Figures S1‐S7, Tables S2‐S5).

**Figure 2:**
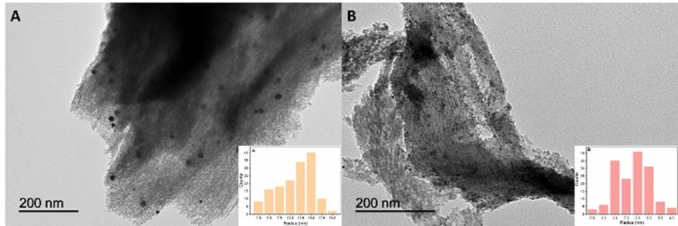
TEM micrographs and the corresponding histograms of catalysts (A) 5Pd/CNi and (B) 5Pd/CNs.

The morphology of representative samples, namely those containing 5 % Pd loading, was analysed by TEM, and the resulting micrographs are presented in Figure 2. Both 5Pd/CNi (Figure 2A) and 5Pd/CNs (Figure 2B) exhibited high uniformity and well‐dispersed palladium nanoparticles supported on a laminar *N*‐doped carbon matrix. Remarkably, the average diameter of the nanoparticles was notably smaller for the sample prepared using Method B. Specifically, 5Pd/CNi exhibited an average radius of 11.3±1.0 nm, whereas 5Pd/CNs displayed a much smaller average radius of 2.6±1.0 nm. These results are consistent with previous reports in the literature and confirm that the solution‐based method can yield superior control over nanoparticle size and morphology.

### Heck‐Mizoroki Reaction

The Heck reaction typically requires relatively high temperatures, and in the literature, polar aprotic solvents with high boiling points have commonly been employed. Solvents such as DMF, DMA, or *N*‐methylpyrrolidone are frequently used for this purpose.^[26]^ However, these solvents are toxic, and pose disposal problems when dissolved in water. In this regard, γ‐Valerolactone (GVL) is an interesting alternative due to its comparable polarity and boiling point (207 °C), along with the additional benefit of being non‐toxic and derived from biomass.^[26]^ Considering such premises, GVL was chosen as solvent in this work. Initial reaction conditions were based on literature reports by Perosa and coworkers, and Strappaveccia et al.[^26^, ^27^] Reaction products were identified by GC‐MS. Secondary products were rarely observed, confirming the high selectivity of these types of cross‐coupling reactions. Quantification analysis of the reaction, to determine the conversion of iodobenzene to the cross‐coupling product (ethyl cinnamate) was achieved using ^1^H‐NMR spectroscopy, with deuterated chloroform as solvent. This approach has been used in an article by Melchiorre et al. (see details in the ESI, Figure S8).^[28]^

### Optimization of Parameters

**Scheme 1:**
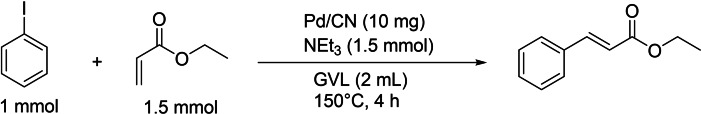
Schematic representation of the investigated Heck‐Mizoroki reaction.

*Catalyst screening*. Initially, a catalyst screening was carried out at 150 °C for 4 h to determine the influence of the Pd loading on the conversion and selectivity of the investigated reaction (Scheme 1). Also, the commercial catalyst Palladium on carbon with a loading of 5 % w/w (5Pd/C) was tested. Under these conditions, all tested catalysts exhibited complete conversion and yielded quantitative results, as can be seen in Figure S9. Therefore, the 1Pd/CNi catalytic system was selected for further optimization, primarily due to its low metal loading and the environmentally friendly nature of Method A. To assess the progress of the reaction in the absence of Pd, two control experiments, one in absence of any catalytic system, the other using the metal‐free nitrogen‐doped carbon support, were conducted. In both cases, negligible conversion was observed.

**Figure 3:**
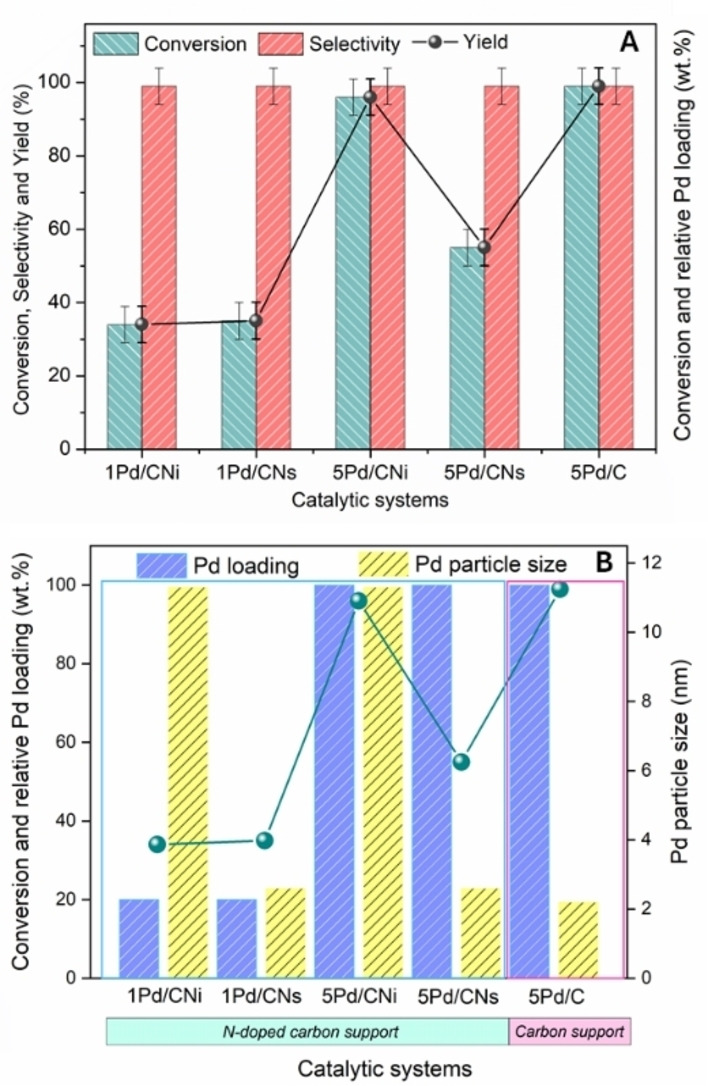
Catalysts screening and its relationship to metal loading and particle size. Please consider that for a clearer illustration metal loading was reported in relative terms, considering that 5 wt.% Pd loading represented 100 %, while 1 wt.% represented 20 %.

Additionally, to gain a more comprehensive understanding of the catalytic behaviour of the synthesized systems, further catalyst screening analyses were conducted under milder conditions (specifically at 80 °C for 4 hours, see Figure S10 and Figure 3). These experiments revealed that for samples with lower metal loading, such as 1Pd/CNi and 1Pd/CNs, regardless of the nanoparticle size, similar conversion values (approximately 35 %) were attained. Conversely, experiments involving a higher metal loading, namely employing the 5Pd/CNi and 5Pd/CNs catalytic systems, yielded contrasting outcomes. Specifically, the 5Pd/CNi sample exhibited higher conversion, reaching up to 96 %, compared to the 5Pd/CNs sample, which achieved only 55 % conversion. It is noteworthy that the catalytic activity of a sample′s active sites depends on various factors, including the metal loading, surface area, and nanoparticle size. While it could be expected that, keeping the metal content constant and with similar surface areas (around 300 m^2^/g in both cases, see ESI, Table S3), a lower nanoparticle size would lead to a higher number of active sites and consequently to a higher catalytic activity, the observed results indicated otherwise. Such outcome suggested that factors beyond particle size were influencing catalytic activity.

For comparison, commercial 5Pd/C was also tested, as it presents a similar particle size to 5Pd/CNs (confirmed by TEM analysis, see Figure S11), but differs in support composition without the presence of nitrogen. Intriguingly, it was found that 5Pd/C exhibited higher catalytic activity, akin to that of 5Pd/CNi. This suggests that not only the support played a crucial role in the catalytic performance, but that there is most likely a combined effect of the metal particle size and the nitrogen dopant species. The presence of nitrogen in the carbonaceous support may lead to Coulombic repulsion between the *p_z_* orbital of nitrogen and electron‐rich moieties, such as nanoparticles or aromatic compounds.^[29]^ Considering this, an explanation for the observed results was formulated, wherein the higher catalytic activity of the commercial catalyst, characterized by smaller nanoparticles, was attributed to the absence of nitrogen in the carbonaceous support, thereby averting repulsive interactions between the reactants and the support surface. In contrast, the favourable catalytic performance observed with the 5Pd/CNi material was associated to the presence of larger nanoparticles, potentially facilitating interactions between active sites and reactants while limiting repulsive interactions with the *N‐*doped support.

Based on these findings, the comparable low conversion rates observed in both the 1Pd/CNi and 1Pd/CNs samples can be attributed to two distinct factors. Firstly, the larger particle size of the 1Pd/CNi sample results in a lower number of available active sites, thereby reducing the conversion efficiency. Secondly, in the case of the 1Pd/CNs sample, the repulsive interactions between the substrate and the support material create an additional hindrance for the catalytic process.^[30]^

#### pH Drift Titration Analysis

**Figure 4:**
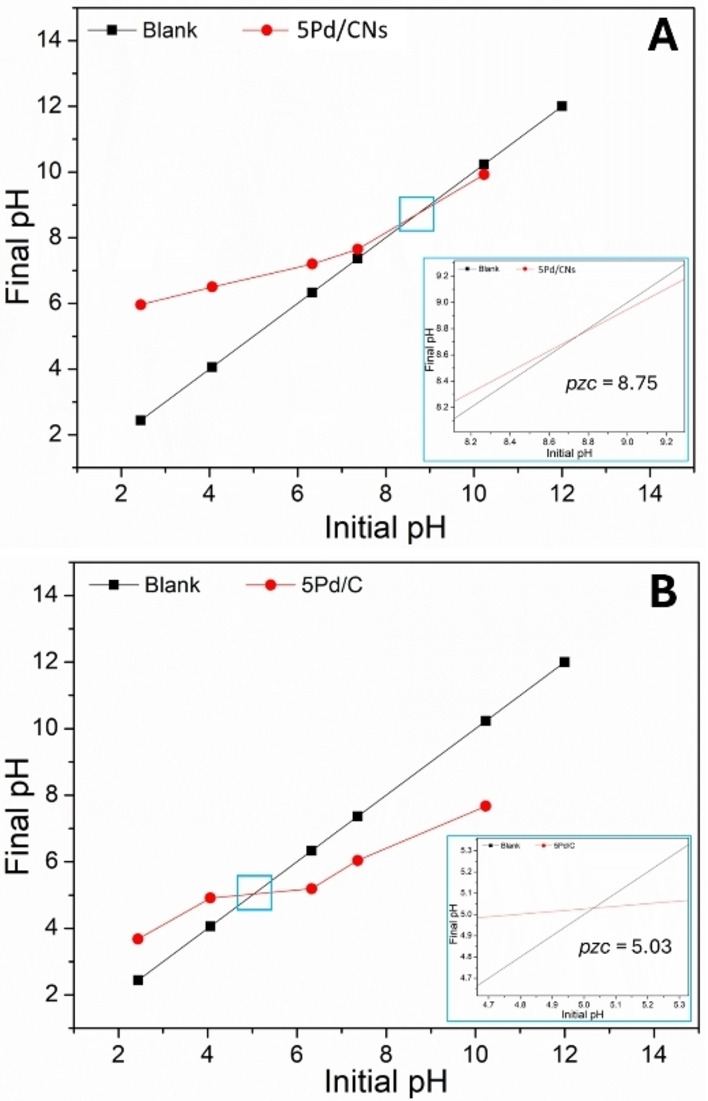
Determination of the PZC of A: 5 %Pd/CNs and B: 5Pd/C utilizing pH drift methods.

To better understand the influence of the support, pH titration experiments were conducted to determine the point of zero charge (*pzc*) for two key samples (Figure 4): 5Pd/CNs and commercial 5 %Pd/C. Both samples had similar palladium loadings (approximately 5 %) and particle sizes (around 1–3 nm) as shown in Figure 3. The determined *pzc* values were 5.03 for 5 %Pd/C and 8.75 for 5Pd/CNs, indicating distinct surface charge characteristics. Notably, when the initial pH is below the *pzc*, the material carries a negative surface charge, while an initial pH above the *pzc* results in a positive charge. In general terms, in a neutral aqueous solution, the 5Pd/CNs sample exhibits a negative surface charge, whereas the commercial 5 %Pd/C sample presents a positive charge.[^31^, ^32^] These results, reflecting varying surface charges based on different support materials, primarily explain the observed differences in catalytic activity between the two samples.

#### Kelvin Probe Atomic Force Microscopy

**Figure 5:**
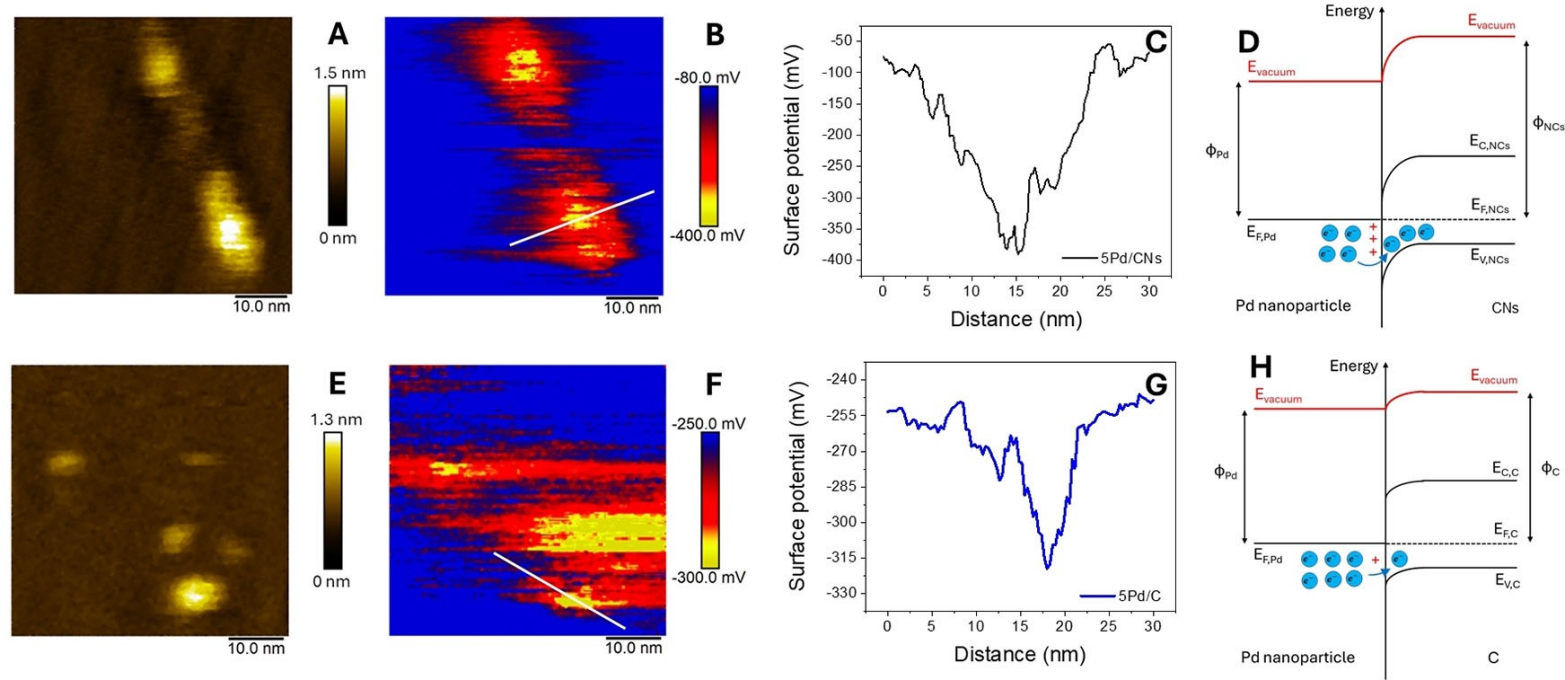
Experimental topography image (A, E), Kelvin probe signal (B, F, C, G) and energy level schemes of Schottky contacts between p‐type support and palladium in presence of thermal equilibrium (D, H) for 5Pd/CNs (up) and 5 %Pd/C (down), respectively.

Kelvin Probe Atomic Force Microscopy (KPFM) is a specific characterization technique that not only allows visualization of the topography of catalytic materials (utilizing atomic force microscopy) but also enables imaging of the surface potential of a material.[^33^, ^34^] This is achieved by coating the AFM tip with a conductive material (Pt−Ir in our case) and applying a V_DC_ voltage (referred to as the Kelvin Probe signal) to neutralize the charge transfer that occurs spontaneously between the conductive AFM tip and the probed surface once electrically connected. When the V_DC_ bias is applied to the sample, the Kelvin probe signal equals the contact potential difference between the tip and the sample, V_CPD_, which is defined as:

$$V_{CPD}=\frac{\varphi_{tip}-\varphi_{s}}{-e}$$

where ‐*e* is the electron elementary charge, $\varphi_{tip}$ and $\varphi_{s}$ are the work functions (WF) of the tip and sample, respectively. Negative values of V_CPD_ imply $\varphi_{tip}$ higher than $\varphi_{s}$ . The contrast of V_CPD_ in KPFM images is directly related to the WF differences on the surface of the sample. To the best of our knowledge, the application of KPFM to palladium nanoparticles supported on *N*‐doped carbons has not been previously reported. Applied to these materials, KPFM provides insight on charge transfer and metal‐support interactions. Specifically, negative Kelvin probe signals indicate a positive electrostatic potential at the tip, suggesting a transfer of negative charge from the metal particle to the underlying support (Figure 5). The observed negative Kelvin signals on Pd particles correspond to positive electrostatic potentials and denote a transfer of negative charge from the noble metal particle to the carbon or *N*‐doped carbon supports. The magnitude of the Kelvin signal varies with the support material, being largest for *N*‐doped carbon compared to the carbon‐based support of the commercial 5 %Pd/C, consistent with known differences in metal‐support interactions. For the 5 %Pd/C material, a difference in Kelvin signal from −250 mV to −300 mV is observed. Moving the tip along the line indicated in Figure 5F, a variation in surface potential from approximately −255 mV on the support to about −320 mV when the tip is placed on the Pd nanoparticle is noticed. This relatively small signal difference indicates a weak charge density polarization, donated from the metal to the support. However, when dopants are introduced into the support, particularly pyridinic and pyrrolic nitrogen acting as *p*‐type dopants, the difference in Kelvin signal in the material ranges from −80 mV to −400 mV. Moving the tip along the highlighted line in Figure 5B shows how the surface potential varies from around −100 mV on the support to about −380 mV when the tip is above the Pd nanoparticle.

This significant variation in Kelvin signal suggests a strong metal‐support interaction with greater electron density delocalization from Pd to the support. This behaviour is summarized and explained in Figure 5D: the presence of *p*‐type dopants leads to stabilization of the valence and conduction bands of the support, lowering the Fermi level and thus increasing the WF difference with the metal, which consequently donates more electrons to the support. As a result, at equilibrium the support will be more negatively charged, with Pd displaying a more electropositive behaviour.

These features could interfere with the Heck reaction during the oxidative addition stage, often the rate‐determining step of cross‐coupling reactions, which is favoured by more electron‐donating metals. This, coupled with a repulsive effect of the negatively charged support towards the substrates, may be most likely the main reasons behind the observed lower conversion of the 5Pd/CNs catalytic material. Although the material 5Pd/CNi also has *p*‐type dopants on the support, the larger nanoparticle size leads to a relatively lower loss of electron density, thus interfering less with the oxidative addition stage. Additionally, any possible electrostatic repulsion from the support is minimized by the greater substrate‐support distance during Pd coordination.

*Influence of the base*. In the Heck reaction, the base facilitates the reductive elimination of HX final step leadings to the regeneration of the catalytically active metallic Pd species.^[35]^ The influence of the nature of the base on the catalytic performance and the reaction progress is shown in Figure S12. Among the bases tested, including triethylamine (NEt_3_), K_2_CO_3_ and Na_2_CO_3_, no significant differences were observed. Therefore, further investigations regarding the base quantity were carried out, in particular using NEt_3_. Although the catalytic cycle consumes the base, which must be introduced in at least stoichiometric amounts relative to the limiting reagent, investigations were also conducted to determine whether the inherent basicity of the support, mainly attributed to the presence of pyridinic N, could potentially offset and decrease the required amount of added base (Figure S12). Unfortunately, no significant influence of the support was observed. In fact, when the reaction was conducted without NEt_3_, no conversion was detected. Instead, the reaction achieved complete conversion when 1 mmol of base was used, so it was chosen as optimal for subsequent tests.

*Temperature optimization*. Subsequently, the optimization of temperature was carried out, as can be seen in Figure S12. The tests were conducted at 50, 75, 100, 110, 125, and 150 °C, achieving quantitative yield when performing the reaction at 125 °C. Non‐significant conversion was detected at 50 °C (<5 %), while at 75 °C the conversion reached 52 %. As a high conversion (93 %) was obtained at 100 °C, a test was also performed at an intermediate temperature of 110 °C; however, no significant differences were observed compared to the test conducted at 100 °C (same conversion of 93 %). The Heck reaction typically requires elevated temperatures, and the results obtained align with those found in the literature.[^26^, ^36^] Therefore, 125 °C was chosen as the temperature for further studies.

*Time optimization*. The study of the reaction time was conducted at 0.5, 1, 2, 3, and 4 h (Figure S12). During the first 30 minutes, almost no conversion was detected (<5 %). Instead, after 1 h a yield of 60 % was achieved. As expected, the conversion gradually increased, reaching values of 83 % and 93 % after 2 and 3 h, respectively. Quantitative conversion was only achieved after 4 h.

**Figure 6:**
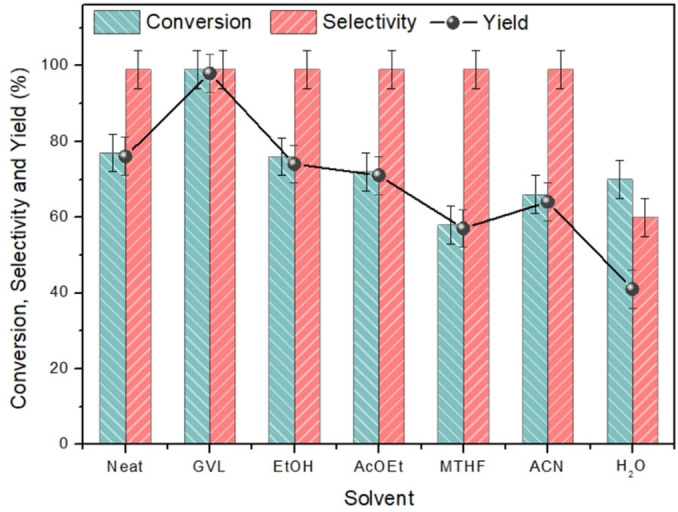
Solvent screening of Heck‐Mizoroki reaction. Iodobenzene (1 mmol), ethyl acrilate (1.5 mmol), NEt3 (1 mmol), 1Pd/CNi (10 mg), 125 °C, 4 h.

*Solvent screening*. After optimizing the reaction conditions, the influence of the solvent on the conversion of iodobenzene was examined (Figure 6). Various alternatives to GVL were tested, all with good polarity and different boiling points. In cases where the solvents boiling point was lower than the required reaction temperature (125 °C), the reactions were conducted in pressurized reactors (autoclaves). Moreover, when feasible based on the solvent signal, the conversion of iodobenzene was assessed using GC‐FID. The results indicated that GVL was the most favourable solvent for this type of cross‐coupling, underscoring its position as a remarkable substitute for hazardous solvents such as *N,N*‐dimethylformamide (DMF) *N,N*‐dimethylacetamide (DMA). The reaction proceeded with conversion values ranging from 60 % to 70 % for all solvents, with polar solvents performing notably well. Among them, ethanol exhibited the highest performance with a conversion rate of 76 %, while less polar solvents, such as methyl‐THF, yielded a conversion rate of 58 %. Considering that the oxidative addition step is rate‐determining, a characteristic that holds true for most cross‐coupling reactions, it′s noteworthy that a solvent with higher polarity can effectively stabilize the highly polarized three‐centre transition state. This, in turn, facilitates and promotes the reaction. In water, despite good conversion (70 %), the selectivity was only 60 % towards ethyl cinnamate due to partial hydrolysis of the ester to the corresponding carboxylic acid (cinnamic acid). Finally, the neat reaction showed promising results with a conversion of 77 %.

### Substrate Scope

Following the establishment of the optimal reaction conditions, investigations into substrate scope were carried out, as shown in Table S6.

Upon analysing the results, it was substantiated that the reactivity of aromatic halides is significantly higher for iodides in comparison to bromo‐ or chloro‐substituted counterparts, a phenomenon commonly observed in cross‐coupling reactions.^[35]^ This discrepancy is attributed to the weaker nature of the I−C bond when contrasted with the C−Br and C−Cl bonds, rendering it more susceptible to cleavage during the initial oxidative addition step. Moreover, there appears to be some steric hindrance imparted by the aryl halide, as evidenced by the superior performance of iodobenzene relative to other disubstituted aryls. Notably, 4‐Iodoanisole exhibited the best results after iodobenzene, achieving a conversion rate of 76 %. Conversely, the lower conversion observed with 4‐iodoaniline might be attributable to the coordinating effect of the amino group, which has the potential to coordinate with Pd, thereby hindering the progression of the catalytic cycle.

### Literature Comparison

The obtained results were compared to other chitin‐based catalytic systems reported in the literature (see Table 1). The reported results are consistent with those of this study, suggesting that catalytic systems derived from seafood industry waste could offer an environmentally friendly alternative for this type of reaction.

**Table 1:** Comparative analysis of the Heck cross‐coupling reaction between iodobenzene and different alkenes, considering recent literature reports.

	Alkene	Catalyst	Solvent	Base	Time [h]	T [°C]	Yield [%]	Ref.
1	Ethyl acrylate	1Pd/CNi	GVL	NEt_3_	4	125	99	This work
2	Methyl acrylate	Pd@CS/PAAS	DMA	NEt_3_	3	110	98	^[37]^
3	*n‐*butyl acrylate	Pd@CS/PAAS	DMA	NEt_3_	3	110	95	^[37]^
4	Methyl acrylate	Pd‐CS/PVA	DMSO	NEt_3_	3	110	95	^[38]^
5	Styrene	Pd/chitin‐Ar	DMF/H_2_O	NEt_3_	10	90	78.8	^[39]^
6	Styrene	PdNP@ChNC	ACN/H_2_O	K_2_CO_3_	24	90	99	^[40]^
7	Ethyl acrylate	10 %‐Pd/C	GVL	NEt_3_	2	125	90	^[26]^

### Suzuki‐Miyaura Reaction

**Scheme 2:**
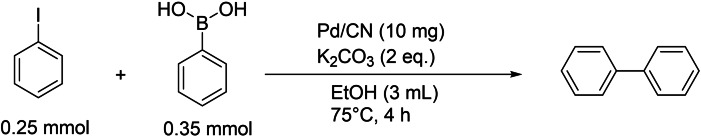
Schematic representation of the investigated Suzuki‐Miyaura cross coupling reaction.

The catalytic systems were additionally tested in the Suzuki‐Miyaura cross‐coupling between iodobenzene and phenylboronic acid (Scheme 2). The initial reaction conditions were based on an article by García‐Suárez et al.^[41]^ Nonetheless, due to challenges associated with low catalyst dispersion, an alternative environmentally friendly solvent was initially selected, namely ethanol. This choice aligns with the recommendations provided in the CHEM21 project′s solvent guide (Chemical Manufacturing Methods for the 21st Century Pharmaceutical Industries).^[42]^ Conducting the reaction in an open vessel under an air atmosphere resulted in the formation of a small amount of the homocoupling product between two phenylboronic acids. However, this was consistently a minor occurrence, emphasizing the catalytic systems’ high selectivity even when the reaction is conducted under such conditions. Quantification was performed using GC‐FID with a calibration curve using an internal standard (mesitylene, 25 μl) for both the product (biphenyl) and the limiting reagent (iodobenzene).

### Parametric Analysis

*Catalyst screening*. Similarly to the Heck‐Mizoroki reaction, catalyst screening was initially conducted to determine the influence of Pd loading on the reaction progress (Figure S13). The reaction, carried out under 75 °C for 4 h, resulted in quantitative yields with all catalytic systems. Two control experiments were performed, one in the absence of catalyst and the other using the *N*‐doped carbonaceous support. However, in both cases, no conversion was observed, confirming the essential role of Pd in promoting these types of reactions. Therefore, for subsequent studies, the material with the lowest catalytic loading, synthesized using the most sustainable protocol (method A), namely 1 % Pd/CNi was chosen.

*Temperature optimization*. Experiments were conducted at 25, 50, and 75 °C (just below the boiling point of ethanol), and results are shown in Figure S14. At room temperature, no conversion was observed, but the difference between 50 and 75 °C was significant, leading to an increase in biphenyl yield from 57 % to 100 %. This result aligns with other studies in the literature, which indicate that 80 °C is the optimal temperature.^[43]^ Despite this temperature being slightly outside the green window (0–70 °C) defined by the pharmaceutical industry in the CHEM21 project, conducting the reaction just below the reflux condition allows for up to a five‐fold reduction in energy consumption. According to the same guidelines, this approach helps meet the operational conditions that a process should aspire for (green flag).^[44]^

*Time optimization*. With these results in hand, a study on reaction time was conducted (Figure S14). After only 30 minutes, a conversion of around 30 % was observed. The plateau was reached after approximately 2 h, with a yield of 92 %, and complete conversion was achieved only after 4 h of reaction.

*Solvent screening*. Generally, in reactions involving compounds with significantly different chemical and physical properties, the choice of solvent is crucial to bring all these substances into contact and allow the diffusion of reagents near the active site of the heterogeneous catalyst.^[36]^ For these reasons, polar aprotic solvents are often used. In Suzuki reactions, the solvent plays a critical role as it can act as a nucleophile (having previously reacted with the base or not) and interact with either the catalyst or the arylboronic acid during the transmetallation step.^[45]^ For this reason, studies on polar protic solvents like H_2_O and alcohols, or mixtures of protic‐aprotic solvents (e. g., DMF/H_2_O), are commonly found in the literature.^[43]^ In this work, ethanol was selected to address these considerations comprehensively, although other solvents were also tested, such as H_2_O, acetonitrile (ACN), methanol, ethyl acetate (AcOEt), methyl‐THF, and GVL. From the results obtained, a noticeable difference is evident between polar protic solvents like ethanol and methanol, which give quantitative yields, compared to the others, which provide yields equal to or less than 10 % (Figure S14). This can be explained by considering the role of the base in the reaction. The carbonate makes ethanol (or methanol) more nucleophilic, which either activates the arylboronic acid or replaces the halide coordinated to Pd, forming less stable species that complete the transmetallation phase. Instead, with the other solvents, this does not occur, and the mild conditions may not be sufficient to overcome the activation barrier of the transmetallation step. Among these results, water provides intermediate yields (40 %). In that case, the reaction product, typically insoluble in water, is brought into solution by diluting the reaction mixture with ethanol. Additionally, during the reaction, unlike with the other solvents, in water, the catalyst does not effectively disperse but remains more aggregated, indicating poor hydrophilicity. In this scenario, the reagents encounter difficulties in diffusing to the interface with the active site located on the catalyst′s surface.

**Figure 7:**
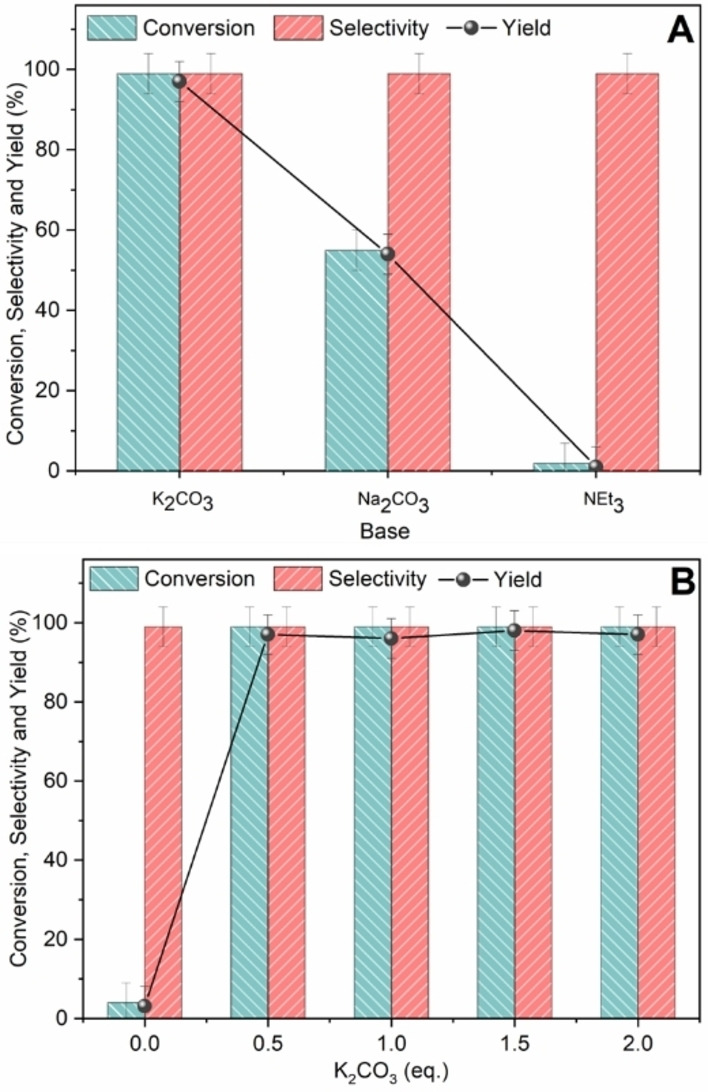
A: Study of the influence of the employed base in Suzuki‐Miyaura cross‐coupling reaction. Iodobenzene (0.25 mmol), phenylboronic acid (1.5 eq.), base (2 eq.), 1Pd/CNi (10 mg), EtOH (3 mL), 75 °C, 4 h. B: Study of the influence of the base concentration in Suzuki‐Miyaura cross‐coupling reaction. Iodobenzene (0.25 mmol), phenylboronic acid (1.5 eq.), 1Pd/CNi (10 mg), EtOH (3 mL), 75 °C, 4 h.

*Base screening*. The role of the quantity and type of base within the Suzuki reaction was investigated. Although the nature of the base does not play a major role as in the Heck cross‐coupling, it was observed that the base is essential in the Suzuki reaction as it can either directly activate the phenylboronic acid to an organoboronate species or activate the protic solvent (alcohol or H_2_O) as nucleophiles. Initially, a study was conducted to determine which of NEt_3_, Na_2_CO_3_, and K_2_CO_3_ yielded the best results under the reaction conditions described above (Figure 7). The results are profoundly different: with K_2_CO_3_, a quantitative yield is obtained, while with Na_2_CO_3_, only 55 % yield is achieved. This behaviour is explained by the lower strength of the ionic pair formed between K^+^ and CO_3_^2−^ compared to Na^+^ and CO_3_^2−^. In this way, the carbonate exhibits higher basicity, promoting the reaction. The results obtained with NEt_3_ were instead unexpected (although very low conversion is also found in the studies of Miyaura and Suzuki) but could be explained by its different coordinating capacity compared to K_2_CO_3_. Assuming that the reaction mechanism involves initial substitution of the halide coordinated to palladium with the base and the subsequent transmetallation with phenylboronic acid, the significantly higher coordinating capacity of the amine can induce a more stabilized species, slowing the catalytic cycle. Once it was established that K_2_CO_3_ is the most suitable base to promote the reaction, tests at different concentrations of the base were performed, as shown in Figure 7. It was found that even when used in sub‐stoichiometric quantities, K_2_CO_3_ can promote quantitative yield. To verify the possible basicity of the support, a test was conducted in the absence of a base. However, similarly to the Heck‐reaction, also in this case low to negligible conversions were reached (yield: 4 %).

### Substrate Scope

With the optimized conditions in hand, a substrate scope was conducted. Various aryl halides and arylboronic acids were tested. The results shown in Table S7 indicate, similarly to the Heck‐Mizoroki reaction, a significant difference in reactivity between iodo‐aryls compared to bromo‐ and chloro‐analogues. These differences are attributed to the weaker halogen‐carbon bond, which favors the initial oxidative addition step. Within iodinated aryls, there are marked differences based on the substituents on the aromatic ring. Since the aromatic ring acts as an electrophilic reagent in cross‐coupling, electron‐donating substituents will hinder the reaction (as seen with 4‐iodoanisole and even more so with 4‐iodoaniline), while electron‐withdrawing substituents will promote the reaction (as observed with 4‐iodoacetophenone or 3‐iodobenzonitrile). For the same aryl halide (iodobenzene), excellent results were obtained by varying the arylboronic acid, with higher reactivity observed for boronic substrates with electron‐donating substituents in the para position on the benzene ring. Regarding selectivity, despite the reaction occurs in the presence of air, less than 5 % of the homocoupling product is obtained, demonstrating that these catalytic systems can effectively control possible side reactions.

### Literature Comparison

The obtained results were compared with other catalytic systems recently reported in the literature. This information is summarized in Table 2.

**Table 2:** Different catalytic systems for Suzuki cross‐coupling reaction between Iodobenzene and phenylboronic acid, employing K_2_CO_3_ as base.

Entry	Catalyst	Solvent	Time [h]	T [°C]	Yield [%]	Ref.
1	1Pd/CNi	Ethanol	4	75	97	This work
2	Pd(TPP)/MB‐H_2_O_2_	H_2_O	4	65	79	^[46]^
3	Pd(TOP)/MB‐1500	H_2_O	4	65	90	^[46]^
4	Pd‐AMP‐Cell@Al_2_O_3_	Ethanol	3	80	72	^[47]^
5	Pd‐AMP‐Cell@Al_2_O_3_	DMF	1	80	95	^[47]^
6	SH−Pd/AC	H_2_O/Ethanol	1	80	99	^[48]^
7	Pd‐PPc‐4	DMF/H_2_O	3	60	99	^[49]^

### Catalyst Recyclability

**Figure 8:**
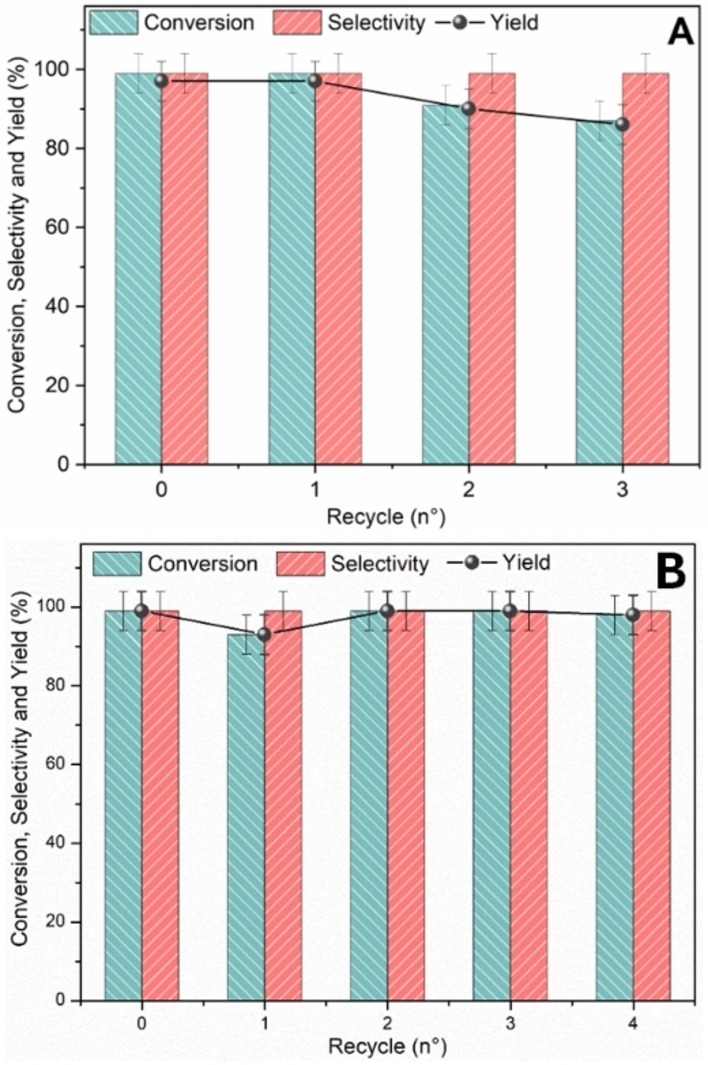
A. Recyclability study of the Heck‐Mizoroki reaction. Iodobenzene (1 mmol), phenylboronic acid (1.5 mmol), NEt3 (1 mmol), 1Pd/CNi (10 mg), GVL (2 mL), 125 °C, 4 h. B. Recyclability study of the Suzuki‐Miyaura reaction. Iodobenzene (0.25 mmol), arylboronic acid (1.5 eq.), K2CO3 (0.5 eq.), 1Pd/CNi (10 mg), ethanol (3 mL), 75 °C, 4 h.

To assess the stability of the 1Pd/CNi catalytic system through multiple cycles, Heck‐Mizoroki cross‐coupling between iodobenzene and ethyl acrylate was carried out under the optimized conditions. After the first reaction, three additional cycles were carried out, achieving excellent conversions, albeit with a slight decrease. Specifically, conversions of 99 %, 91 %, and 87 % were obtained for the 1st, 2nd, and 3rd cycles, respectively (Figure 8 A). This could be attributed to several factors, including metal leaching, sintering of the metallic nanoparticles, or adsorption of organic moieties on the catalyst surface. These phenomena may be more pronounced at higher temperatures.

Hence, investigations were carried out on the catalyst stability under milder conditions, akin to those in the Suzuki‐Miyaura reaction. Notably, as depicted in Figure 8B, negligible alterations in conversion were noted over four reaction cycles. These findings suggest that the variations in catalytic performance observed in the Heck‐Mizoroki reaction were likely linked to the impact of elevated temperatures.

### Post‐Characterization Analysis

In any case, to gain a deeper understanding of these results, the recycled catalyst, referred to as R‐1Pd/CNi, was subjected to analysis to investigate any potential changes in its crystalline structure, morphology, chemical composition, and surface properties. To achieve this, XRD, XPS, and N_2_ physisorption analyses were performed. All details are reported in the Supporting Information File (Figure S15–19, Table S8). Overall, in the recycled catalyst after the Heck reaction, a decrease in the XRD signal was observed, which, along with a reduction in surface area (measured via N_2_ physisorption, which also confirmed mesoporous behavior), was attributed to the idea that organic molecules adsorb onto the catalyst during the reaction, leading to the occlusion of smaller pores. Additionally, XPS analysis showed a slight oxidation of Pd on the catalyst surface, with the ratio of Pd^0^ to Pd^II^ changing from approximately 3 : 1 to around 1 : 1.

### Mechanochemical Approach

**Figure 9:**
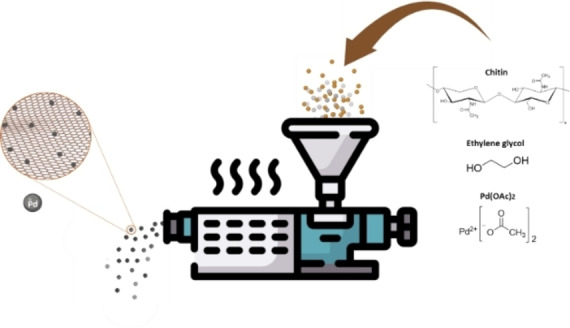
Schematic representation of the mechanochemically assisted synthesis of the catalytic materials.

With a view to establishing more sustainable synthetic protocols for these catalytic materials, an extrusion process was designed (Figure 9), employing chitin, palladium acetate, ethylene glycol and, in some instances, a base. The experimental parameters were selected based on prior experience of our research group for the mechanochemical synthesis of nanomaterials.[^23^, ^50^, ^51^] Ethylene glycol served as a green reducing agent, based on recent reports in the literature.^[52]^ The resulting material in the form of small cylindrical extrudates (approximately 1 mm in diameter, See Figure S20) was collected and used, either directly or after a thermal treatment at 500 °C under N_2_ atmosphere, as catalyst for the Suzuki‐Miyaura cross‐coupling.

In a modified approach, Na_2_CO_3_ (53 mg, 0.5 mmol) was introduced, so as to intensify the protocol by having the base directly present in the catalyst and potentially avoiding adding it to the reaction mixture. The four resulting materials were designated as P‐5Pd/CN‐ex and 5Pd/CN‐ex for catalysts obtained without and with thermal treatment in the absence of a base, respectively. Meanwhile, P‐5Pd/CN‐ex‐Na_2_CO_3_ and 5Pd/CN‐ex‐Na_2_CO_3_ were assigned to catalysts obtained without and with thermal treatment in the presence of Na_2_CO_3_, respectively.

### Materials Characterization

The structure of the samples was assessed by XRD. The XRD patterns prior to thermal treatment and of the thermally‐treated samples are shown in Figures S21A and S21B, respectively.

The P‐5Pd/CN‐ex and P‐5Pd/CN‐ex‐Na_2_CO_3_ materials exhibited the characteristic diffraction peaks of chitin at approximately 10, 20, 21, 23, and 26°, corresponding to the (020), (110), (120), (101), and (130) crystallographic planes, respectively (Figures S21A).[^53^, ^54^] A broad peak observed between 20° and 30° likely indicated the presence of amorphous carbon formed during the mechanochemical synthesis at 190 °C. Moreover, a signal at 39.2° indicates the successful reduction of Pd (II) leading to the formation of Pd(0) nanoparticles. As shown in Figure S21B for the thermally‐treated samples, the broad signal at approximately 24.0° was attributed to the (002) crystallographic plane associated with stacked graphene‐like sheets, indicating the formation of amorphous carbon.^[55]^ The sharp and well‐defined peaks at 40.1, 46.6, and 68.1° were attributed to the (111), (200), and (220) crystallographic planes of palladium, respectively, confirming the presence of Pd(0).[^56^, ^57^, ^58^]

The thermally treated samples, both with and without the base, were further characterized in terms of particle size by applying the Scherrer equation to the XRD diffraction patterns (Table S9).[^59^, ^60^] The calculated particle sizes were (14.9±0.5) nm and (10.3±0.5) nm for 5Pd/CN‐ex and 5Pd/CN‐ex‐Na_2_CO_3_, respectively. The presence of Na_2_CO_3_ in the mechanochemical synthesis of the catalyst appears to have influenced the dimension of the nanoparticles, leading to smaller particle size. These results suggest that a repulsive effect of sodium cations or the coordination of carbonate with palladium, both of which prevent possible agglomeration of nanoparticles during extrusion, result in a reduction in nanoparticle size. However, further studies are required to confirm these hypotheses.

**Figure 10:**
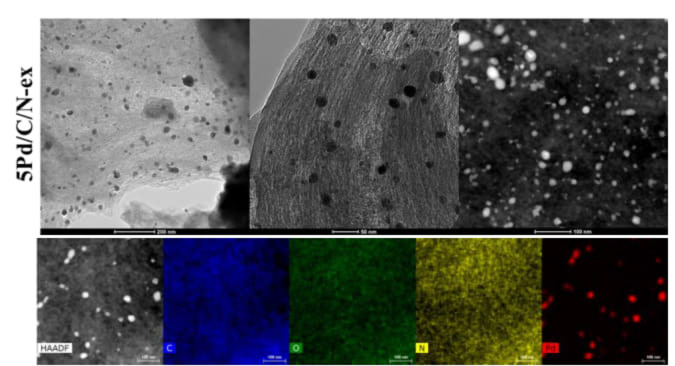
Representative HRTEM, STEM and EDX‐mapping micrographs of 5Pd/CN‐ex.

Further investigations on the morphology of the catalytic systems were conducted by HRTEM (Figure 10 and Figure S22). The micrographs showed that both samples exhibited highly uniform and well‐dispersed palladium nanoparticles supported on a lamellar *N*‐doped carbon matrix. In addition, the average diameter of the nanoparticles was calculated, confirming the results obtained through the Scherrer equation. Indeed, the values obtained from the TEM analysis are (16.6±1.0) nm and (10.0±1.0) nm, respectively, for P‐5Pd/CN‐ex and P‐5Pd/CN‐ex‐Na_2_CO_3_. In addition, EDX‐mapping measurements confirmed the presence of carbon, nitrogen, oxygen, and palladium in both catalytic samples, with all elements being uniformly distributed in each case (Figure 10 and Figure S22). In addition, for the sample 5Pd/CN‐ex‐Na_2_CO_3_, a homogeneous distribution of sodium can also be observed, deriving from the incorporation of Na_2_CO_3_ into the material.

The textural properties of the samples, including surface area, pore volume, and pore size, were examined by N_2_‐physisorption. Both materials showed physisorption isotherms type IV with type II adsorption hysteresis, indicative of the formation of a mesoporous material. Figure S23 displays a representative isotherm of 5Pd/CN‐ex. The surface area was calculated using BET equation, while pore volumes and mean pore size diameter was calculated using the BJH equation. Both samples exhibited a remarkably high surface area of 452 and 498 m^2^ g^−1^ for 5Pd/CN‐ex and 5Pd/CN‐ex‐Na_2_CO_3_, respectively, demonstrating a significant improvement of the textural properties compared to the materials prepared using a solvent (5Pd/CNi and 5Pd/CNs showed surface areas around 300 m^2^/g). Finally, the range of pore diameter around 5.0 nm further confirmed the presence of mesoporous solids. In summary, these characterization studies validated the mechanochemical protocol for the preparation of supported metal nanoparticles on mesoporous *N*‐doped carbon‐based matrices.^[61]^ Finally, the palladium loading, determined by ICP‐OES, was around 4 mg/g_catalyst_ for both samples. The data are summarised in Table S9.

**Figure 11:**
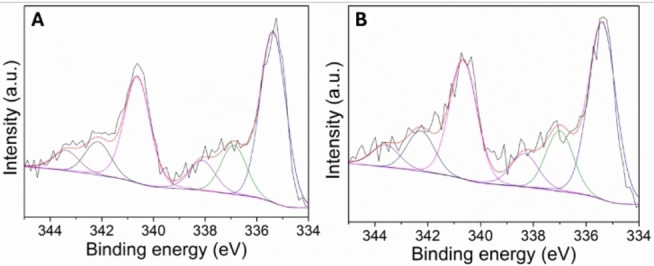
High resolution XPS spectra of the Pd 3*d* XPS regions of 5Pd/CN‐ex (A) and 5Pd/CN‐ex‐Na2CO3 (B).

XPS analyses are shown in Figure S24. The spectra generally exhibit similar characteristics to those of materials obtained through the more conventional methods A and B. More interesting, an assessment of the chemical composition of the palladium entities on the catalysts surface, specifically for 5Pd/CN‐ex and 5Pd/CN‐ex‐Na_2_CO_3_, was conducted based on the Pd 3 *d* core level spectra. Notably, these spectra displayed marked differences from all other samples (refer to Figure 11A and B). In this case, the Pd 3*d* signals were deconvoluted into six distinct contributions. Four of these contributions correspond to the previously mentioned ones, located at (335.1±0.2) eV and (340.3±0.2) eV referred respectively to the doublet Pd 3*d*_5/2_ ‐ Pd 3*d*_3/2_ of Pd(0), and at (336.2±0.2) eV and (341.5±0.2) eV associated respectively to the doublet Pd 3*d*_5/2_ ‐ Pd 3*d*_3/2_ of Pd(II) oxide. In addition to these signals, two additional contributions can be observed, located at (343.6±0.2) eV and (338.3±0.2) eV, indicating the presence of Pd−*N* bonds.^[62]^

It is interesting to note that the mechanochemical synthesis promotes the formation of Pd−N bonds, unlike the conventional syntheses using methods A and B. This enhanced interaction between the metal nanoparticles and the support is likely to result in increased catalyst stability against leaching phenomena, a persistent challenge in heterogeneous catalysis.

XPS spectra were collected for the materials synthesized via mechanochemistry without undergoing the subsequent thermal treatment, labelled as P‐5Pd/CN‐ex and P‐5Pd/CN‐ex‐Na_2_CO_3_. The spectra can be found in the ESI (Figure S25). The only notable differences were observed in the C 1*s* core level spectra, where there is a change in the relative intensity between the signals associated with C−C/C=C bonds from graphitic and/or aromatic carbon, C−OH, C−N/C−O, and C=O moieties. Indeed, before the thermal treatment, there is a prevalence of signals from C−N/C−O species and a 1 : 1 ratio between the signals of C−C/C=C species and C=O species. As expected, after the thermal treatment, the contributions from carbonyl carbons and C−N/C−O bonds decrease, conversely the signals related to graphitic and/or aromatic carbon increase. The N 1*s* core level spectra also showed differences, exhibiting a single signal most likely associated with the amide nitrogen of chitin, with a binding energy of (399.5±0.2) eV. Finally, the Pd 3*d* core level spectra exhibit the same four signals as the catalysts prepared using conventional methods. However, for the material obtained using Na_2_CO_3_, the ratio of Pd(0)/Pd(II) signals is shifted more towards Pd(0), indicating a probable promoter effect of the base in the reduction of the metal precursor during the mechanochemical treatment.

### Catalytic Activity

Catalytic activity was tested under mechanochemical solvent‐free conditions on the Suzuki‐Miyaura reaction between iodobenzene and phenylboronic acid. The reactions were carried out in a twin‐screw extruder under semi‐continuous‐flow conditions. Both the catalytic systems synthesized using conventional protocols (methods A and B) and those prepared through mechanochemical methods were tested for comparison. The aim was to unravel whether the catalytic coupling mechanism changes under solvent‐free mechanochemical conditions, as compared to operating in solution. In this direction, a plausible mechanism has been proposed in the literature by Pentsak and coworkers.^[63]^ The authors conducted a mechanochemical cross‐coupling between various aryl halides and arylboronic acids under solvent‐free conditions using a ball milling. The proposed mechanism entails the generation of trace quantities of water, formed by the condensation of three molecules of arylboronic acid. Subsequently, the water can react with the base to form the hydroxide anion, which promotes the transmetallation step by either activating the arylboronic acid or by activating the palladium catalyst by replacing the halide. Additional water can derive from the conversion of boric acid formed during the catalytic cycle under basic conditions into potassium borate, with the release of water, which re‐enters the catalytic cycle. This proposed mechanism is supported by experimental evidence.^[63]^

*Mass loading*. Building upon the conditions previously optimized batch reactions, the initial mechanochemical experiment focused on varying the mass loaded into the extruder. While this parameter may not hold particular significance in conventional batch reactions using solvents, it plays a pivotal role in mechanochemical processes. The optimal amount of mass within the extruder could enhance the mixing efficiency and boost the mechanical energy involved, thus promoting more efficient contact between the reagents. Consequently, after conducting some preliminary tests that demonstrated promising outcomes within an hour, the quantities of reagents were scaled‐up from 0.25 (50 mg) to 2 mmol (400 mg) for the limiting reagent (iodobenzene), while preserving the relative molar ratios of the other reagents. Using 5Pd/CNi as catalytic system, two different catalyst loading were tested: 20 mg and 40 mg. However, only slightly higher conversions were obtained for the latter case (an increase from 52 % to 58 % conversion). On the contrary, when maintaining a constant catalyst quantity of 40 mg and doubling the amount of reagents introduced (from 2 mmol (400 mg) to 4 mmol (800 mg) of iodobenzene), the conversion reached 93 % within just 1 h at 80 °C. These preliminary results already highlight the advantages of the mechanochemical protocol, which include the elimination of solvents and the increased productivity compared to batch reactions. The catalytic systems 5Pd/CN‐ex and P‐5Pd/CN‐ex were also tested for comparison. After 1 h at 80 °C, an improvement in performance was observed when using 2 mmol of the limiting reagent, as compared to the 5Pd/CNi catalytic system, resulting in 73 % and 81 % conversions for the P‐5Pd/CN‐ex and 5Pd/CN‐ex catalysts, respectively. Similarly to the 5Pd/CNi catalyst, when it comes to the mechanochemically‐prepared materials, increasing the mass loading by doubling the amount of reagents, while keeping constant the catalyst quantity, led to a conversion rate of 95 %. These results align with the previously observed trend. A summary of the results is shown in Table 3.

**Table 3:** Mass loading study. Phenylboronic acid (1.5 eq.), K_2_CO_3_ (2 eq.), 80 °C, 1 h, twin screw rotation velocity 50 RPM. Selectivity >99 %.

Entry	Iodobenzene [mmol, mg]	Catalyst [mg]	Conversion [%]	Yield [%]
1	2, 408	5Pd/CNi [20]	52	51
2	2, 408	5Pd/CNi [40]	58	57
3	4, 816	5Pd/CNi [40]	93	92
4	2, 408	P‐5Pd/CN‐ex [40]	73	72
5	2, 408	5Pd/CN‐ex [40]	81	80
6	4, 408	5Pd/CN‐ex [40]	95	93

**Figure 12:**
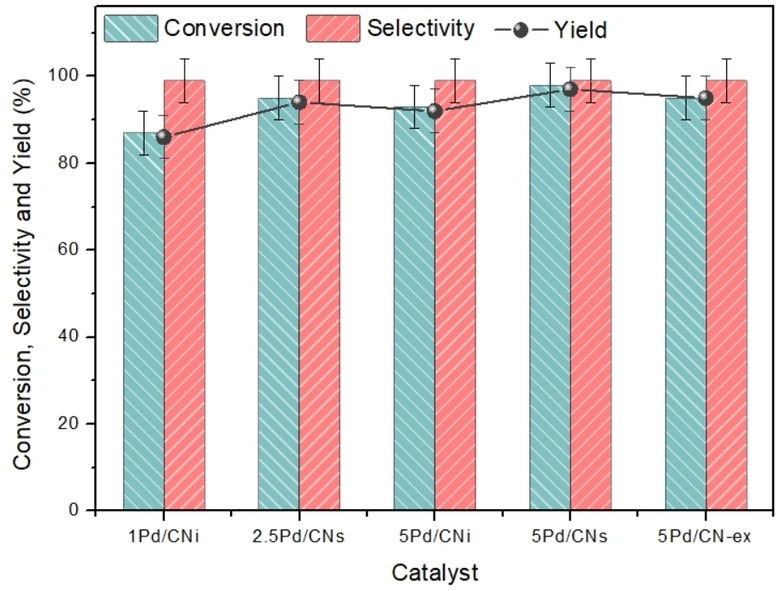
Catalyst screening of Suzuki‐Miyaura reaction using mechanochemical protocol. iodobenzene (4 mmol), phenylboronic acid (1.5 eq.), K2CO3 (2 eq.), catalyst (40 mg), 50 rpm of velocity of twin screw, 80 °C, 1 h.

*Catalyst screening*. Having defined the optimal loading of limiting reagent as 4 mmol, a catalyst screening was conducted to identify the most productive system. The reaction was conducted in the extruder at 50 rpm and 80 °C for 1 h. Satisfactory results were reached for all the tested catalytic systems, namely 1Pd/CNi, 2.5Pd/CNs, 5Pd/CNi, 5Pd/CNs, and 5Pd/CN‐ex, which yielded conversions of 87 %, 95 %, 93 %, 98 %, and 95 %, respectively (Figure 12). Based on these conversions, it may appear that the 5Pd/CNs system performs best. However, a comparison of conversion considering the amount of Pd, indicates the following productivity of the catalytic systems: 8.70, 3.80, 1.96, 1.86, and 1.90 mol/(g_Pd_h) for the 1Pd/CNi, 2.5Pd/CNs, 5Pd/CNi, 5Pd/CNs, and 5Pd/CN‐ex samples, respectively. Based on this analysis, the 1Pd/CNi catalytic system emerges as the more efficient. Additionally, with the aim of investigating whether the quantity of Na_2_CO_3_ introduced in the synthesis of the 5Pd/CN‐ex‐Na_2_CO_3_ catalyst could replace the K_2_CO_3_ base typically used in the reaction, tests with the catalytic systems 5Pd/CN‐ex‐Na_2_CO_3_ and P‐5Pd/CN‐ex‐Na_2_CO_3_ were conducted, in the absence of additional base. Unfortunately, these tests did not yield significant conversions (<10 %).

*Time and Temperature optimization*. Finally, the operational conditions were optimized, specifically focusing on temperature, residence time, and rotation speed. Tests conducted at 50, 100, and 150 rpm gave identical results. Time and temperature were optimized (Figure S26) on the 1Pd/CNi catalytic system by running three experiments at 0.5, 1, and 2 h, resulting in conversions of 58 %, 87 %, and 93 %, respectively. Considering productivity, the optimal residence time was found to be 1 h. Regarding temperature, experiments were conducted at room temperature (25 °C), 50 °C, and 80 °C, resulting in conversions of 5 %, 15 %, and 87 %, respectively. In this case, the importance of temperature was evident, with 80 °C yielding the best results.

It is noteworthy that following the mechanochemical reaction, the product was separated from the solid catalysts using a minimal amount of solvent, specifically ethanol. The solid catalysts were then recovered via filtration, enabling their reuse, while the solvent was recovered using a rotavapor and recycled for subsequent tests. The Heck reaction was also conducted in the extruder; however, the presence of all liquid reagents limited the application of the mechanochemical approach (where the presence of solids critically influences the involved mechanical forces) and no conversion was observed. In any case, the catalyst 5Pd/CN‐ext was also tested in Heck reaction in solution, showing quantitative conversion and full selectivity toward the cross‐coupling product under the above‐mentioned optimized condition (10 mg of 5Pd/CN‐ext, 1 mmol of iodobenzene, 1.5 mmol of ethyl acrylate, and 1 mmol of NEt_3_, 125 °C, 4 hours). Recycling tests of the catalyst were also performed, suggesting excellent stability (see supporting information, Figure S27). This was corroborated by ICP‐OES analysis of the catalytic material, which showed leaching below 1 % of the initial metal concentration in the sample.

*Comparison with the state‐of‐the‐art*. The optimal results obtained with the synthesized catalytic systems were compared to other case studies found in recent literature regarding Suzuki‐Miyaura cross‐coupling using mechanochemical protocols. These observations are summarized in Table 4.

**Table 4:** Comparative analysis with recent literature reports about Suzuki cross‐coupling reaction using a mechanochemical protocol.

Entry	Catalyst	Reactor	Additive liquid (LAG)	Time ^[h]^	Yield [%]	Productivity [mol/gPdh]	Ref.
1	1Pd/CNi	Extruder	No	1	87	8.70	This study
2	Pd‐Chitin‐500	Extruder	No	1	81	0.81	Previous work^[61]^
3	Pd sphere	Ball milling	Ethanol	0.50	99	0.0005	^[64]^
4	Pd layer	RAM*	Ethanol	1	90	0.018^[a]^	^[65]^
5	Pd(OAc)_2_	Ball milling	H_2_O	0.08	57	2.14	^[66]^

[a] the estimation of productivity was calculated considering the deposition of all palladium contained in the electrolyte solution employed for the electroplating of milling vessel (2 g/L), taking into account the total volume of the vessel (25 mL) as amount of solution. *Resonance Acoustic Mechanochemistry.

## Experimental

All chemicals employed during the reactions and the synthesis of catalysts were commercially available compounds sourced from Sigma‐Aldrich and are presented in Table S1. If not otherwise specified, reagents and solvents were employed without further purification. Gas Chromatography‐Mass Spectrometry (GC‐MS, Electron Ionization (EI), 70 eV) analyses were performed on a HP5‐MS capillary column (L =30 m, Ø =0.32 mm, film =0.25 mm). GC (flame ionization detector; FID) analyses were performed with an Elite‐624 capillary column (L =30 m, Ø =0.32 mm, film =1.8 mm). All reactions were performed in duplicate to verify reproducibility.

Quantification analysis to determine conversion values was performed by ^1^H‐NMR in the Bruker Avance III HD 400 WB equipped with a 4 mm CP/MAS probe, employing deuterated chloroform. Furthermore, the identification of the obtained products was performed by GC‐MS in the Agilent 7820 A GC/5977B High Efficiency Source (HES) MSD.

### Synthesis of Catalytic Materials

*N*‐doped carbon‐supported Pd nanoparticles were synthesized with different amounts of Pd precursor, to achieve loadings of 1 %, 2.5 % and 5 % (w/w). In order to reach these concentrations during the synthesis, 0.1, 0.25 and 0.5 mmol of palladium (II) acetate (Pd(OAc)_2_) were employed, respectively. According to the procedure in the article of Rodriguez‐Padron et al.^[58]^, catalytic systems were obtained employing two different protocols: impregnation and solution methods, labelled respectively as procedure A and B.

*Impregnation method*. Firstly, the proper amount of Pd(OAc)_2_ was dissolved in 2‐propanol (15 mL) and subsequently, chitin (5 g) was added into the solution. The materials were left to age overnight at room temperature and further oven dried at 100 °C under vacuum. Finally, the samples were thermally treated at 500 °C (heating rate was 5 °C/min) for 1 h under N_2_ flow (10 Ml min^−1^). The catalytic samples achieved by this procedure were labelled as Pd/CNi.

*Solution method*. The proper amount of Pd(OAc)_2_ was dissolved in 2‐propanol (60 mL), together with EDTA (1 g). Subsequently, chitin (5 g) was added to the mixture, which was kept under stirring for 9 h at 80 °C under reflux. The suspension was filtered and the so‐obtained solid was dried at 100 °C overnight and, finally, heated at 500 °C for 1 h, according to the conditions previously described. The catalytic sample achieved by this procedure was labelled as Pd/CNs.

The resulting materials were ground to powder (particle size <200 μm) and stored in the oven (70 °C, 15 mbar) until further use. The yield of the obtained materials was ca. 35±5 %, based on the total weight of chitin and the metal precursor used.

### Characterisation of Catalytic Materials

The crystalline structure of the samples was investigated by X‐ray diffraction (XRD) in a D8 Advance diffractometer from Bruker® AXS, using the X‐ray source of the Cu Kα radiation, coupled to a LynxEye detector, and monitoring the 2θ within 8–80° at a rate of 0.08° min^−1^.

The chemical composition on the surface of the solids was examined using X‐ray Photoelectron Spectroscopy (XPS) with a Physical Electronics VersaProbe II Scanning XPS Microprobe. This equipment uses a monochromatic X‐ray Al−Kα radiation source under a vacuum of 10^−7^ Pa. The binding energies were calibrated to the C 1*s* peak from adventitious carbon at 284.8 eV. High‐resolution spectra were recorded using a concentric hemispherical analyser at a constant energy pass of 29.35 eV over a 200 μm diameter analysis area. The pressure in the analysis chamber was maintained below 5×10^−6^ Pa. Data acquisition and analysis were performed using PHI ACCESS ESCA−F V6 software. A Shirley‐type background was subtracted from the signals, and the recorded spectra were analyzed with Gauss‐Lorentz curves to determine the binding energy of the atomic levels of different elements more accurately.

The textural properties, i. e., surface area, the volume of the pore and pore size, were determined from N_2_ physisorption at −196 °C, performed in an ASAP 2000 instrument from Micromeritics®. The samples were outgassed at 120 °C for 2 h. The specific surface areas were calculated by the BET method; the pore volumes were calculated from adsorption isotherms and the pore size distributions were estimated using the Barrett, Joyner and Halenda (BJH) algorithm available as built‐in software from Micromeritics (Micromeritics Instrument Corporation (Norcross, GA, USA)).

SEM‐EDX images were acquired in a JEOL‐SEM JSM‐7800 LV scanning microscope. Transmission electron microscopy (TEM) was performed to observe the size and shape of the particles in a JEOL 2010 operated at an acceleration voltage of 200 kV and with a FEI Tecnai G2 system, equipped with a charge‐coupling device (CCD) camera. Samples were ground and then suspended in ethanol, followed by dipping of a holey‐carbon coated copper grid of 300 mesh which was left to dry under air for a few minutes prior to recording.

The exact amount of Pd presented in the catalytic systems was obtained through ICP‐OES analysis carried out in the Avio 550 Max ICP‐OES Optical Emission Spectrometer.

Measurements of surface potential were obtained by Frequency Modulation‐Kelvin Probe Force Microscopy (FM‐KPFM), using a Dimension Icon atomic force microscope (Bruker, Germany) in ambient conditions. We used a PtIr‐coated AFM tip on a cantilever with spring constant and resonant frequency of 3 N/nm and 75 kHz, respectively (SCM‐PIT−V2, Bruker, Germany). Frequency and amplitude of the modulation VAC signal were set at 2 Hz and 500 mV, respectively. During acquisition, the external VDC bias to neutralize the surface potential was applied to the sample.

### Catalytic Experiments

Catalytic experiments were carried out in sealed test tubes. In a typical Heck‐Mizoroki cross coupling reaction, 10 mg of the catalyst was added, followed by iodobenzene (1 mmol, 112 μl), ethyl acrylate (1.5 mmol, 160 μl), and triethyl amine (1 mmol, 140 μl) in the stated order. Subsequently, GVL (2 mL) was introduced into the mixture. The solution was then sealed and stirred at 125 °C for 4 h. After the reaction, the catalyst was separated from the solution through filtration. Quantification analysis to determine conversion values was performed by ^1^H‐NMR, employing deuterated methanol. Furthermore, the identification of the obtained products was performed by GC‐MS in the Agilent 7820 A GC/5977B High Efficiency Source (HES) MSD.

In a typical Suzuki‐Miyaura cross coupling reaction, 10 mg of the catalyst was added, followed by potassium carbonate (K_2_CO_3_, 0.5 mmol, 70 mg), iodobenzene (0.25 mmol, 26 μl) and phenylboronic acid (0.35 mmol, 43 μl), in the stated order. Subsequently, ethanol (3 mL) was introduced into the mixture. The solution was then sealed and stirred at 80 °C for 4 h. After the reaction, the catalyst was separated from the solution through filtration. Quantification analysis to determine conversion and selectivity values was performed by gas chromatography (GC) using a flame ionization detector (FID). The measurements were accomplished in the Agilent 6890 N gas chromatograph (capillary column HP‐5, 60 mL min^−1^ N_2_ carrier flow, 20 psi column top head pressure). The identification of the obtained products was performed by GC‐MS in the Agilent 7820 A GC/5977B High Efficiency Source (HES) MSD.

### Mechanochemical Approach: Experimental

#### Synthesis of Catalytic Materials

The materials were synthesized using a mechanochemical extrusion method. This involved the use of chitin (5 g), Pd(Ac)_2_ (0.5 mmol), and ethylene glycol (15 mL). The mixture was extruded in a ZE 12 HMI Twin‐Screw Extruder (Three Tec, Seon, Switzerland) at a temperature of 200 °C and a speed of 50 rpm. The resulting material was then dried in an oven at 100 °C under vacuum overnight before being used in catalytic tests. Additionally, a thermal treatment was performed at 500 °C for 1 h under a flow of N_2_ (10 mL/min) for further comparison. The heating rate for this process was 5 °C.

The synthesis process was also carried out with the addition of Na_2_CO_3_ (0.5 mmol) under the previously described conditions.

#### Catalytic Experiments

A typical CF‐Mechanochemical Suzuki‐Miyaura cross‐coupling reaction was carried out to evaluate the performance of the catalysts. The reaction was conducted under continuous flow conditions using a ZE 12 HMI extruder from Three Tec. The reaction involved iodobenzene (2 mmol), PhB(OH)_2_ (3 mmol), K_2_CO_3_ (4 mmol), and the chosen catalysts (40 mg). The mass loading, temperature, time of reaction, and speed of rotation of extruder were optimized. The conversion and selectivity were determined using GC‐FID after an extraction with a minimum amount of ethanol, while the product structures were identified using GC‐MS.

## Conclusions

In a context where green chemistry and process sustainability are gaining increasing importance, this work proposes utilizing chitin, derived from seafood waste, as a precursor for the preparation of catalyst support materials in line with a circular economy perspective. Three synthetic methods for incorporating palladium into the carbon‐nitrogen matrix were proposed and evaluated based on their nanoparticle size control and environmental impact. The catalytic systems were tested in two cross‐coupling reactions, Heck‐Mizoroki and Suzuki‐Miyaura. An important finding worth highlighting from this study is that it was found that not only particle size, but also a combination of metal particle size and nitrogen dopant species in the carbonaceous support, likely influenced catalytic performance significantly. Furthermore, the use of a novel mechanochemical approach played a pivotal role in this study, as it was utilized for both catalyst preparation as well as for the cross‐coupling reactions. Specifically, a Suzuki reaction between iodobenzene and phenylboronic acid was conducted using a tailored mechanochemical protocol, offering a sustainable and efficient alternative to conventional solution‐based methods.

## Supporting Information Summary

The authors have cited additional references within the Supporting Information.[^14^, ^53^, ^56^, ^65^, ^66^, ^67^, ^68^, ^69^]

## Conflict of Interests

The authors declare no conflict of interest.

1

## Supporting information

As a service to our authors and readers, this journal provides supporting information supplied by the authors. Such materials are peer reviewed and may be re‐organized for online delivery, but are not copy‐edited or typeset. Technical support issues arising from supporting information (other than missing files) should be addressed to the authors.

Supporting Information

## Data Availability

The data that support the findings of this study are available from the corresponding author upon reasonable request.
