# Supplementary material for: Upcycling of Chitin to Cross‐Coupling Catalysts: Tailored Supports and Opportunities in Mechanochemistry
Source: ChemSusChem. 2024 Oct 21;18(1):e202401255. doi: 10.1002/cssc.202401255 (PMC11696199; doi:10.1002/cssc.202401255)
Supplement: Supplementary file 1 — Supporting Information [file CSSC-18-e202401255-s001.pdf]

ChemSusChem

Supporting Information

Upcycling of Chitin to Cross-Coupling Catalysts: Tailored Supports and Opportunities in Mechanochemistry

Oscar Trentin, Daniel Ballesteros-Plata, Enrique Rodríguez-Castellón, Leonardo Puppulin, Maurizio Selva, Alvise Perosa,* and Daily Rodríguez-Padrón*

Supporting Information

UPCYCLING OF CHITIN TO CROSS-COUPLING CATALYSTS: TAILORED SUPPORTS AND OPPORTUNITIES IN MECHANOCHEMISTRY

Oscar Trentin,[a] Daniel Ballesteros-Plata,[b] Enrique Rodríguez-Castellón,[b] Leonardo Puppulin,a Maurizio Selva,[a] Alvise Perosa,[a]* Daily Rodríguez-Padrón[a]*

In this study, chitin derived from shrimp shells was utilized in the development of Pd-based heterogeneous catalysts for cross-coupling reactions. Various methods, including mechanochemistry via extrution, were employed to synthesize supported Pd nanoparticles on N-doped carbons. Comprehensive characterization using multiple techniques revealed that the catalytic performance was significantly impacted not just by the particles size; rather, it was influenced by the interplay between the metal particles size and the presence of nitrogen dopant species within the carbonaceous support. The catalytic behavior of the samples was evaluated in cross-coupling reactions, considering both batch and semi-continuous flow solvent-free conditions, yielding quantitative yields and a productivity of 8.7 mol/(gPdh).

Contents

1. Experimental details 1

2. Materials Characterization 2

3. Catalytic activity 8

4. Catalyst recyclability 13

5. Characterization analysis of mechanochemically prepared materials 15

1. Experimental details

Table S1. Chemicals employed in this work.

REAGENTS	MOLECULAR WEIGHT (u.m.a.)	SOLVENTS	MOLECULAR WEIGHT (u.m.a.)
Iodobenzene	204.02	H_2O	18.02
4-iodoacetophenone	246.05	Methanol	32.04
4-iodoaniline	219.03	Ethanol	46.07
4-iodoanisole	234.04	γ-Valerolactone	100.12
3-iodobenzonitrile	229.02	Acetonitrile	41.05
Bromobenzene	157.01	Ethyl acetate	88.11
3-Bromotoluene	171.04	Methyl-THF	86.13
4-Bromotoluene	171.04	**BASES**	**MOLECULAR WEIGHT (u.m.a.)**
4-chloroiodobenzene	238.46	Triethyl amine	101.19
4-chlorotoluene	126.59	Potassium carbonate	138.21
4-chloronitrobenzene	157.56	Sodium carbonate	105.99
Phenylboronic acid	121.93	**OTHER**	**MOLECULAR WEIGHT (u.m.a.)**
p-tolilboronic acid	135.96	Chitin	221.21*
m-tolylboronic acid	135.96	2-propanol	60.10
4-metoxyphenylboronic acid	151.96	ethylene glycol	62.07

Methylacrylate	86.09	$Pd(OAc)_2$	224.50
Ethylacrylate	100.12	EDTA	292.24

*Referred to molecular weight of the strucural unit N-acetilglucosamine

2. Materials Characterization

The crystal structure of the materials was examined by XRD (Figure S1). Notably, all the XRD patterns of the materials all displayed common signals: a broad reflection around 24.0°, indicating the presence of amorphous carbon, particularly on the (002) crystallographic plane associated with stacked graphene-like sheets.[55] All the samples also displayed sharp and well-defined signals at 39.9°, 46.5°, and 67.9° corresponding to the (111), (200), and (220) planes, respectively, of Pd(0) with a face-centred cubic crystal structure.[67] The progressively less intense palladium-related signals was consistent with a decreasing Pd loading. The XRD patterns of the Pd/CNs materials exhibited slightly broader reflections than Pd/CNi, mostly noticeable in the sample with a higher metal loading. This observation suggests the formation of smaller metal nanoparticles in the samples obtained by method B compared to those obtained by method A.

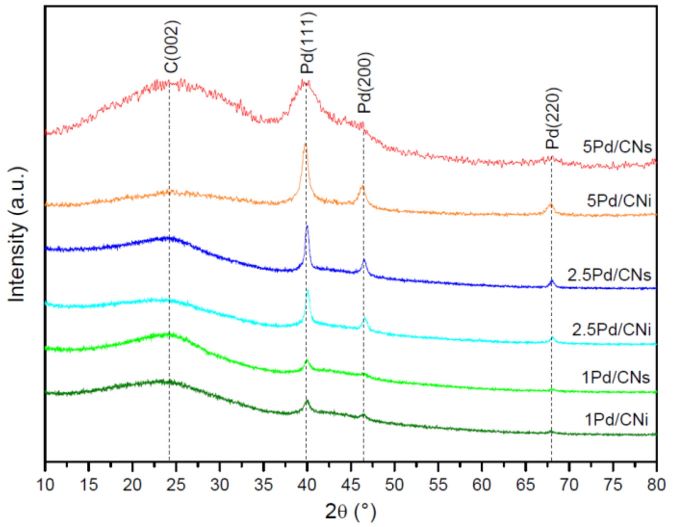

Figure S1. XRD patterns of the catalytic systems obtained with methods A (impregnation) and B (solution).

Additionally, crystallite strain and size were evaluated with XRD using the Rietveld refinement analysis (Table S2).

Table S2: Results of Rietveld refinement from X-ray powder diffraction analysis

Material	Crystallite (rms) Strain/ %	Crystallite Size (nm)
**1Pd/CNi**	0,332	14.7
**5Pd/CN-ex**	0,162	17.5

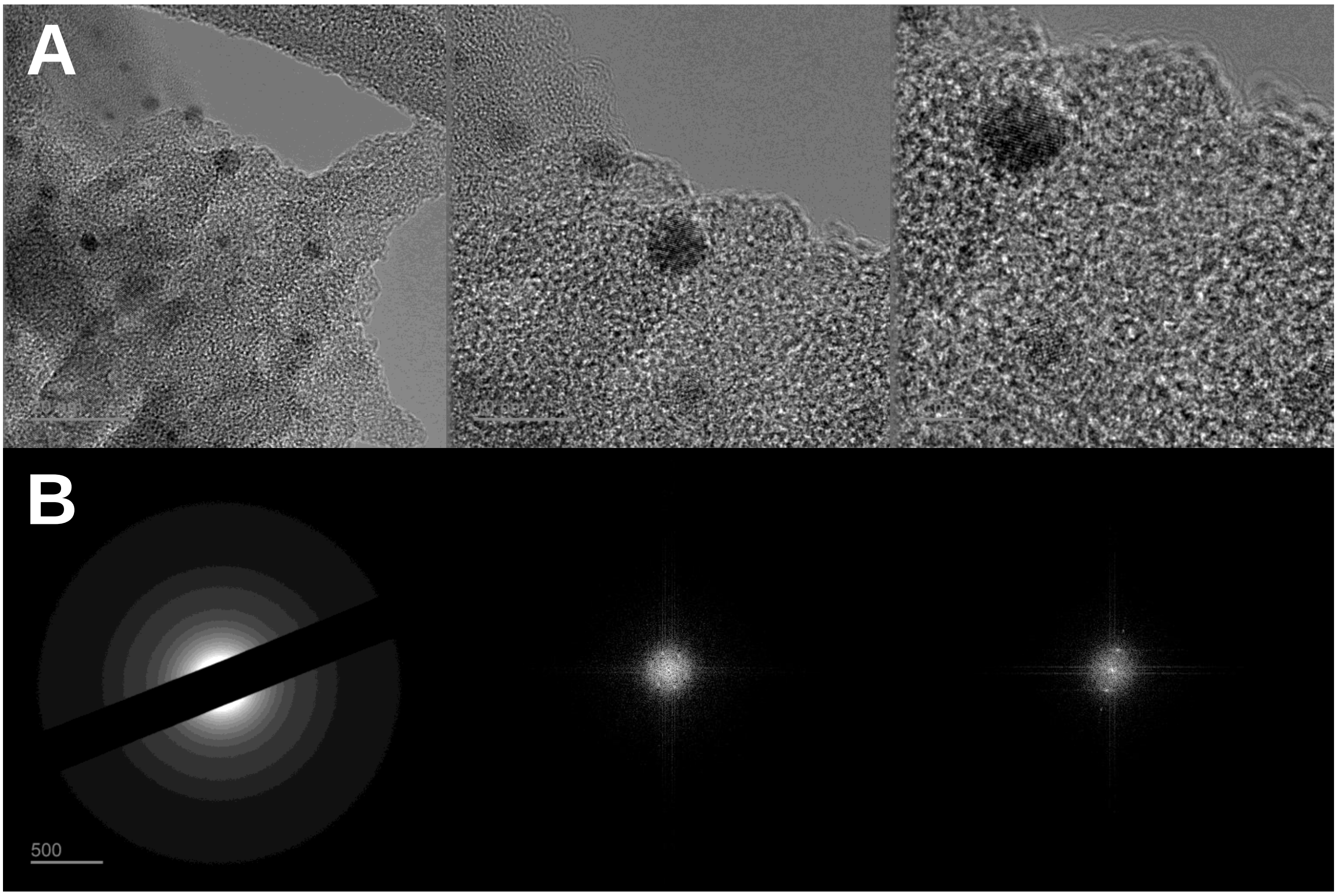

Figure S2. A: HR-TEM micrographs and B: SAED analysis of 1%Pd-N/Ci sample.

Further insight on the morphology and elemental composition of the samples were gained by SEM-mapping (Figure S3), which confirmed the successful incorporation of palladium entities into the *N*-doped carbon matrix. SEM-mapping confirmed the presence of carbon, nitrogen, oxygen, and palladium in both catalytic samples, all elements being uniformly distributed in each case. Additionally, SEM images of 5Pd/CNi and 5Pd/CNs samples, as shown in Figure S3A and S3F, revealed a 3D-irregular arrangement with porosity and a rough morphology.

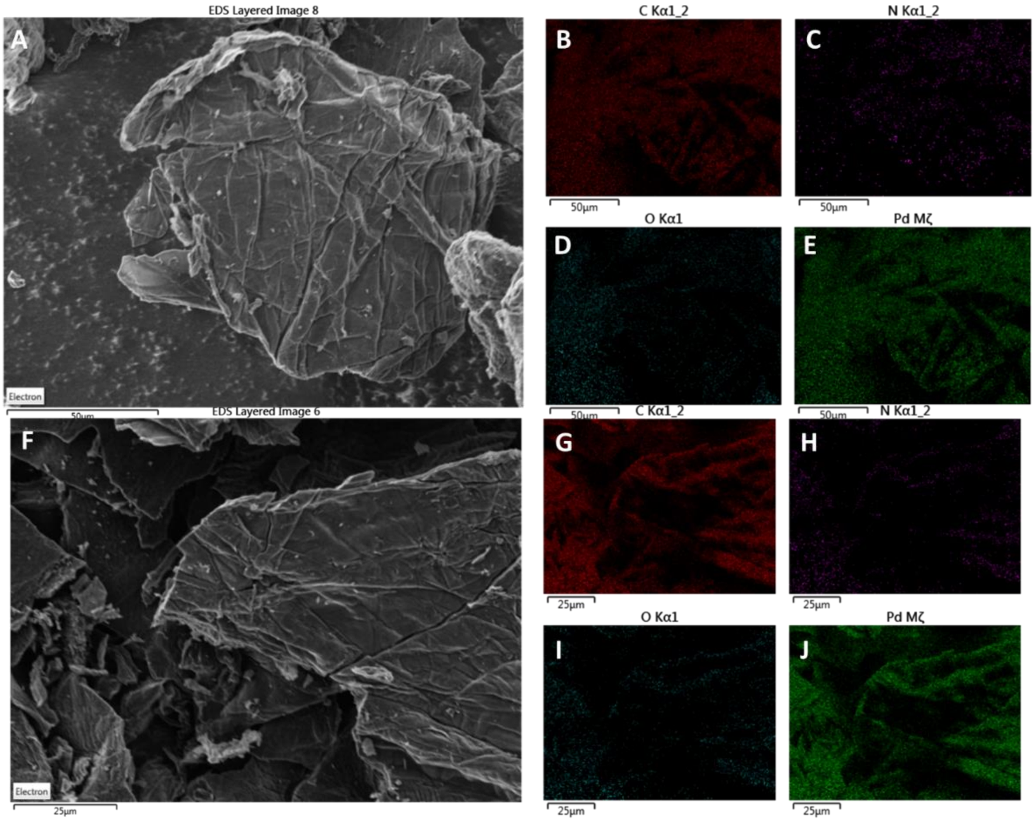

Figure S3: SEM-mapping micrographs of **5Pd/CNi** and **5Pd/CNs** materials. SEM images of **5Pd/CNi** (A) and**5 Pd/CNs** (F). SEM-mapping results of **5Pd/CNi** for carbon (B), nitrogen (C), oxygen (D) and palladium (E). SEM-mapping results of **5Pd/CNs** for carbon (G), nitrogen (H), oxygen (I) and palladium (J).

The textural properties of the samples were examined by N_2 physisorption measurements. To determine whether and how porosity, surface area, and pore size vary with different catalytic loadings, we conducted a study using materials with the lowest and highest loadings—specifically, 5% and 1% Pd. Additionally, to explore potential differences determined by the synthetic methodology of the catalyst, materials obtained by the impregnation and the solution methods were also considered. Firstly, as can be observed in Table S4, all the examined samples exhibit average pore diameters between 3.6 and 6 nm, attributable to mesoporous materials.[58, 68, 69] This classification is further confirmed by experimental physisorption profiles that fit the typical Type IV isotherms associated with mesoporous materials, (Figure S4). In this study the pore volume and the average pore size diameter were calculated employing the Barret-Joyner-Halenda (BJH) model, which considers not only the Kelvin radius but also the monolayer thickness, giving a more realistic response.

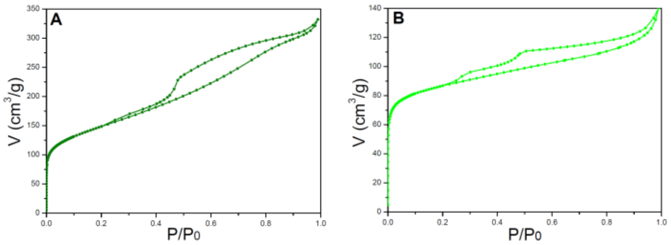

Figure S4. Representative N_2 physisorption isotherms of catalytic systems 1Pd/CNi obtained with method A (Figure 4A) and 1Pd/CNs obtained with B (Figure 4B).

The 5Pd-N/Ci and 5Pd-N/Cs catalysts exhibit similar average pore diameters (4.3 nm and 4.0 nm, respectively), surface area (300 m^2/g and 311 m^2/g, respectively), and pore volume values (0.25 m^3/g and 0.28 m^3/g, respectively) (Table S3, entry 2 and 3). For comparison, chitin was treated under similar calcination conditions, and the textural properties of the resulting *N*-doped carbon are also provided in Table S3, entry 1. The comparison suggests that the textures of the samples were primarily influenced by the carbon-nitrogen matrix and

the calcination protocol, while the metal and its method of incorporation had a minimal impact. The 1Pd/CNi and 1Pd/CNs catalysts were instead rather different. Specifically, 1Pd/CNi displayed smaller average pore sizes, larger surface area and pore volume (3.6 nm, 526 m^2/g, and 0.48 cm^3/g, respectively). In contrast, the 1Pd/CNs catalytic system exhibits larger average pore sizes while maintaining a higher surface area with a lower pore volume (6 nm, 402 m^2/g, and 0.19 cm^3/g, respectively). Examining the average pore size in the 1Pd/CNi and 1Pd/CNs systems, it can be postulated that in the case of catalysts synthesized using Method A, the Pd nanoparticles diffusion into the smaller pores is hindered by the limited solvent volume. Instead, they tend to preferentially deposit within the larger pores. As a consequence, there is no pore occlusion, but rather an overall reduction in pore size.
In any case, these results demonstrated that, in general, the incorporation of Pd leads to an increased surface area of the material, compared to the metal-free counterpart. Recent studies have reported that the presence of Pd nanoparticles on a graphene-like support is responsible for creating nanoholes on the graphene sheets, thereby positively impacting the overall surface area and textural properties.[70] A similar hypothesis could hold in this case, in order to explain the higher surface area of the samples modified with 1 wt.% of palladium. However, this enhancing effect on the surface area is compensated when higher palladium loading (5% Pd materials) is used, most likely due to the partial occlusion of the pores. Regardless, the materials retained excellent textural properties, which could further favour their catalytic performance as will be discussed in subsequent sections.

Table S3: Textural properties obtained by N_2-Physisorbtion of different synthesised catalytic system.

**Material**	**S_{BET} (m^2/g)**	**D_{BJH} (nm)**	**V_{BJH} (cm^3/g)**
**C-N**	320	3.9	0.35
**5Pd/CNi**	300	4.3	0.25
**5Pd/CNs**	311	4.0	0.28
**1Pd/CNi**	526	3.6	0.48
**1Pd/CNs**	402	6.0	0.19

* S_{BET}: specific surface area was calculated using the Brunauer-Emmett-Teller (BET) equation. D_{BJH}: mean pore size diameter was calculated using the Barret-Joyner-Halenda (BJH) equation. V_{BJH}: pore volumes were calculated using the Barret-Joyner-Halenda (BJH) equation.

All the examined samples exhibit mesoporous behaviour, as suggested by the N_2 physisorption isotherms. However, the presence of micropores is not discounted. According to the t-plot method (Table S4 and Figure S5), a significant contribution of mesopores was confirmed, while also accounting for the presence of micropores in the sample. It is important to note that for the scope of the investigated catalytic reactions, mesopores are particularly relevant, as they provide pathways that enhance the diffusion of reactants and products, reducing diffusion limitations and improving the overall reaction rate. Their larger size, in comparison with micropores, prevents blockage by larger molecules, making them particularly useful in reactions involving bulky substrates or intermediates.

Table S4. t-plot analysis of 1Pd/CNi

Micropore volume (cm^3/g)	0.068
Micropore area (m^2/g)	152
External surface area (m^2/g)	374
Total surface area (BET) (m^2/g)	526

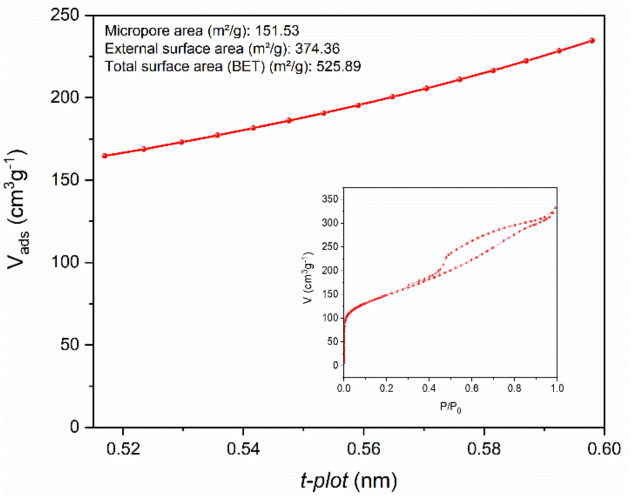

Figure S5. t-Plot analysis of 1Pd/CNi

The chemical composition and surface properties of a catalytic material wield considerable influence over heterogeneous catalysis processes. To understand the chemical nature and elemental composition on the surface of the prepared catalysts, XPS analyses were performed, and the results are illustrated in Figure S6 and Figure S7. As for physisorption, representative materials obtained through both Method A and B were analyzed, with Pd loadings of 1% and 5%. XPS spectra consistently exhibited the presence of carbon, oxygen, nitrogen, and palladium on the surface of all catalytic materials. Specifically, the C 1*s* core level spectra of the materials modified with 5 wt.% palladium loading (Figure S6A, S6E) were deconvoluted into five contributions situated at (284.3 ± 0.2) eV, (285.7 ± 0.2) eV, (287.2 ± 0.2) eV, (288.6 ± 0.2) eV and (290.0 ± 0.2) eV, attributed to C–C/C=C bonds from graphitic and/or aromatic carbon, C–OH, C–N/C–O, C=O and COO^- species, respectively. Conversely, the latter contribution was absent in the case of the 1 wt.% palladium samples, exhibiting only four contributions attributed to C–C/C=C bonds from graphitic and/or aromatic carbon, C–OH, C–N/C–O, and C=O (Figure 5A and 5E). This observation suggests the likelihood of carbon dioxide formation resulting from the decomposition of the organic matrix, a phenomenon likely induced by the higher palladium content present in the samples with a 5 wt.% palladium loading.

The N 1*s* core level spectra (Figure S6B, S6F, 7B and 7F) showed two main bands typically attributed to pyridinic and pyrrolic nitrogen species, at (398.4±0.2) eV and (400.3±0.2) eV. It is worth noting that these *N*-functionalities, particularly pyridinic groups, could serve as active sites in base-catalysed reactions, such as CO_2 fixation.[15] Additionally, the presence of nitrogen could enhance the incorporation and dispersion of metal nanoparticles. Interestingly, graphitic *N*-groups were not found on the surface of these materials.

The O 1*s* core level spectra of the samples, shown in Figure S6C, S6G, 7C and 7G for the 5Pd-N/Ci, 5Pd-N/Cs, 1Pd/CNi and 1Pd/CNs samples, respectively, were deconvoluted into two main contributions. These components, located at (531.2±0.2) eV and (533.2±0.2) eV, are attributed to O-Metal bonds in metal oxides and to the presence of adsorbed H_2O in the catalyst surface, respectively.

Finally, the chemical nature of palladium entities on the catalyst surface was evaluated employing the Pd 3*d* region of the XPS spectra (as shown in Figure S6D, S6H, 7D and 7H). The signals were deconvoluted into four contributions located at (335.1 ±0.2) eV, (336.2±0.2) eV, (340.3±0.2) eV and (341.5±0.2) eV. In particular, the signals at approximately (335.1 ±0.2) eV and (340.3±0.2) eV were associated with the doublet Pd $3d_{5/2}$ and Pd $3d_{3/2}$ of Pd(0); while the band shoulders contributions located at binding energy values of (336.2±0.2) eV and (341±0.2) eV indicated the presence of Pd(II) species of palladium oxide on the catalyst surface. By analysing the XPS data in conjunction with the XRD results, it becomes evident that the synthesized nanoparticles primarily consist of a metallic Pd(0) core, while the surface exhibits partial oxidation.

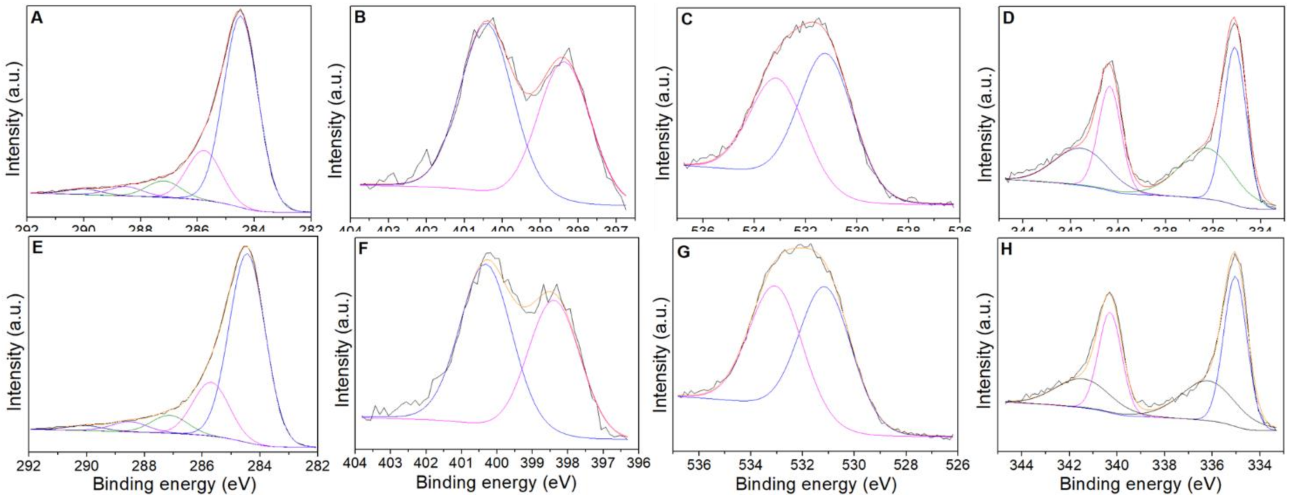

Figure S6: XPS spectra of **5Pd/CNi** (A-D), **5Pd/CNs** (E-H). For each catalytic system, it is represented in this order the C 1*s* (A, E), N1*s* (B, F), O 1*s* (C, G) and Pd 3*d* (D, H) XPS regions.

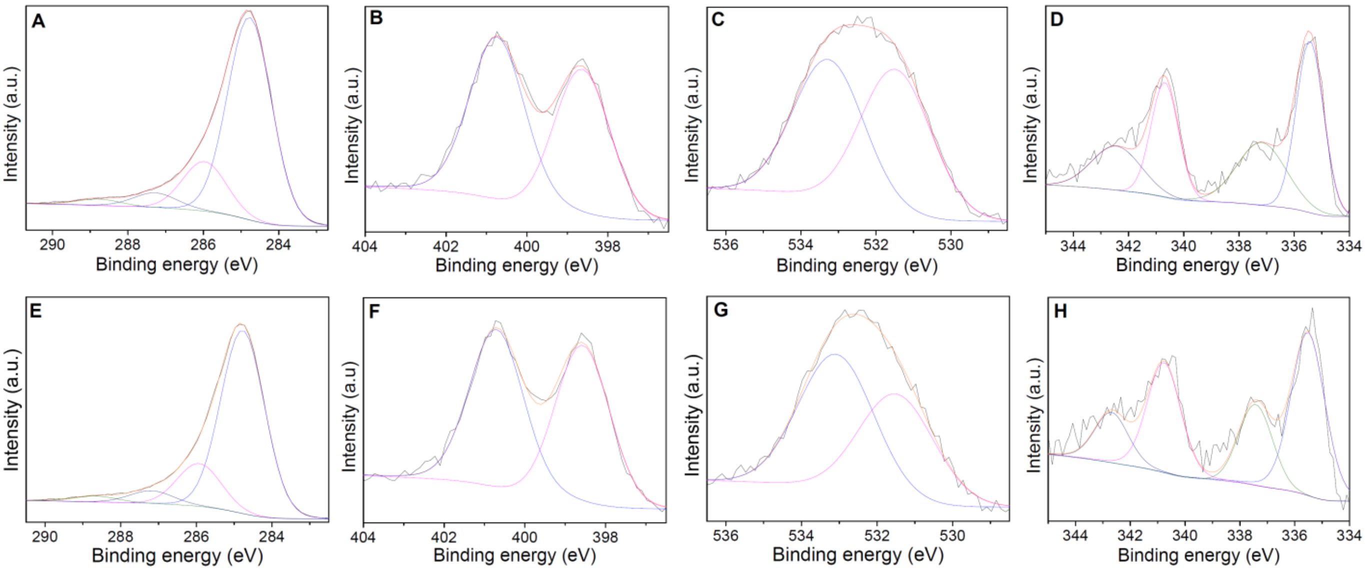

Figure S7. High resolution XPS spectra of 1Pd/CNi (A-D), 1Pd/CNs (E-F). For each catalytic system, it's represented in this order the C 1s, N 1s, O 1s and Pd 3d XPS regions.

XPS quantification was carried out to ascertain the metal content on the catalyst surface, and the findings are detailed in Table S5. Furthermore, ICP measurements were conducted to determine the palladium concentration in the bulk samples, as also documented in Table S5. The weight percentage of total palladium found for the bulk material, as confirmed by ICP-OES results, was consistent with the expected percentages based on the amounts of metal and carbon precursors employed in each synthetic methodology (A and B). Notably, the palladium concentration on the surface, as assessed via XPS, was slightly higher in comparison to the ICP-OES data. These results likely indicate a dispersion of the metal entities within the materials, with a higher concentration residing on the surface of the carbonaceous materials.

Table S5. XPS Binding energy and Palladium concentration according to XPS and ICP-OES results.

**Sample**	**Pd(0) $3d_{5/2}$/eV**	**Pd(II) $3d_{5/2}$/eV**	**Pd wt.% (XPS)**	**Pd wt.% (ICP-OES)**

**5Pd-N/Ci**	335.1	336.4	7.5	5.4
**5Pd-N/Cs**	335.1	336.2	7.6	4.9
**1Pd-N/Ci**	335.1	336.4	1.5	1.1
**1Pd-N/Cs**	335.1	336.2	1.6	1.1

3. Catalytic activity

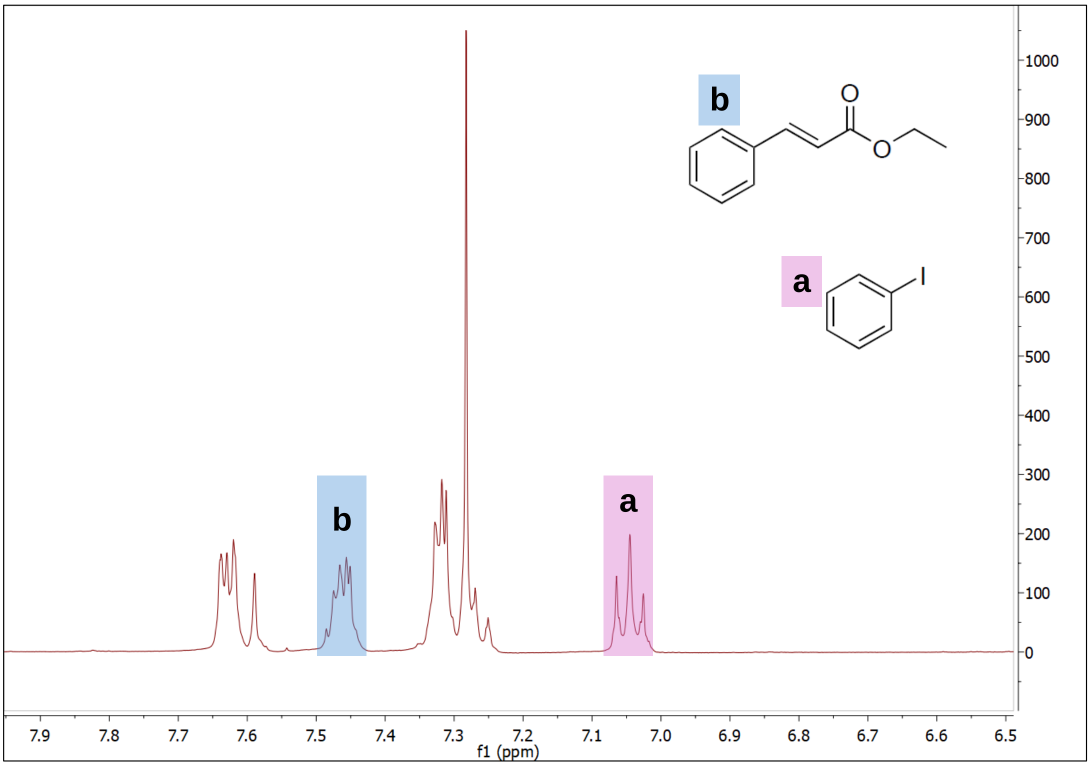

Figure S8: Characteristics signals of ethyl cinnamate and iodobenzene in H[1]-NMR spectra.

By analyzing ^{1}H-NMR spectra, a characteristic signal of iodobenzene at around 7.5 ppm (labeled as *b*) and one characteristic signal of ethyl cinnamate at around 7.1 ppm (labeled as *a*) were identified, allowing the calculation of conversion using the equation 1 displayed below. For the yield, a known quantity of mesitylene was used as a standard, and the equal relaxation time (and therefore signal) of protons with different chemical environments was exploited, following the procedure previously described by Rigo et al. (equation 2), properly modified for the reaction under consideration.[71]

$$\text{Conversion (\%)}=\left(\frac{\int b}{\int a+\int b}\right)\times 100$$

Equation S1: *a*: area of characteristic signal of iodobenzene; *b*: area of characteristic signal of ethyl cinnamate

$$\text{Yield (\%)}=100\times\frac{\left(\frac{3\times N_{std}\times \text{AHa}}{5\times \text{AHs}}\right)}{N_0}$$

Equation S2: N_{std}: amount of internal standard (mesitylene, mol); AHa: integral of the area corresponding to the signal of product (5H); AHs: integral of the area corresponding to the signal of internal standard (3H); N_0: amount of iodobenzene at t_0 (mol).

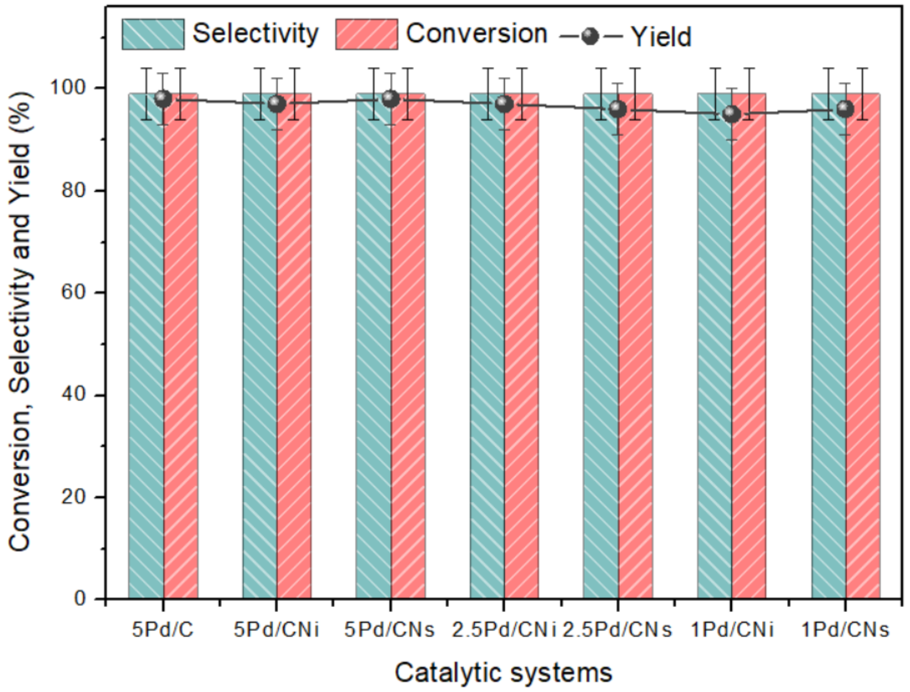

Figure S9: Catalyst screening of Heck-Mizoroki cross-coupling reaction. Iodobenzene (1 mmol), ethyl acrilate (1.5 mmol), NEt_3 (1.5 mmol), catalyst (10 mg), GVL (2 ml), 150 °C, 4 h.

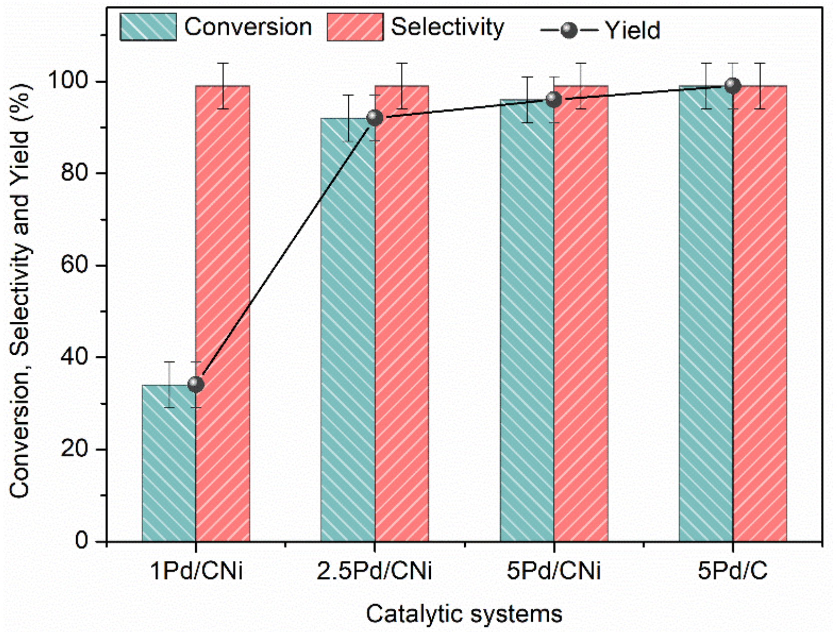

Figure S10: Catalyst screening of Heck-Mizoroki cross-coupling reaction. Iodobenzene (1 mmol), ethyl acrilate (1.5 mmol), NEt_3 (1.5 mmol), catalyst (10 mg), GVL (2 ml), 80 °C, 4 h.

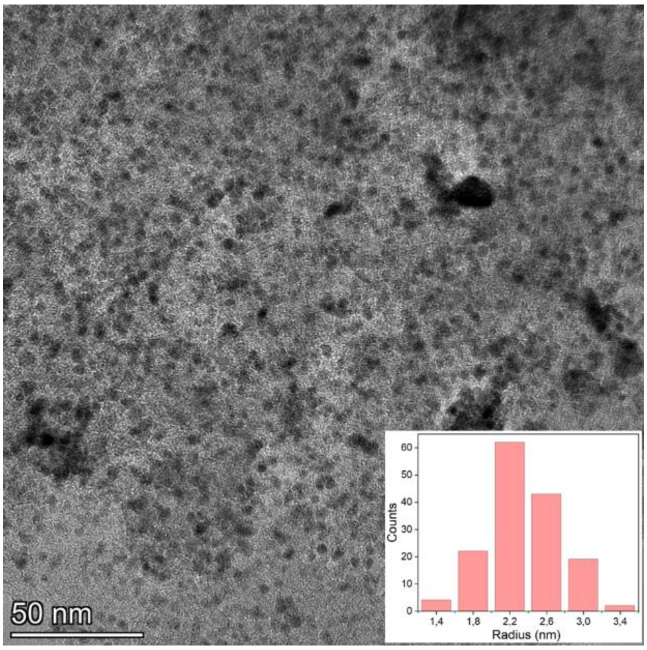

Figure S11: TEM micrograph and the corresponding histogram of commercial catalyst 5Pd/C.

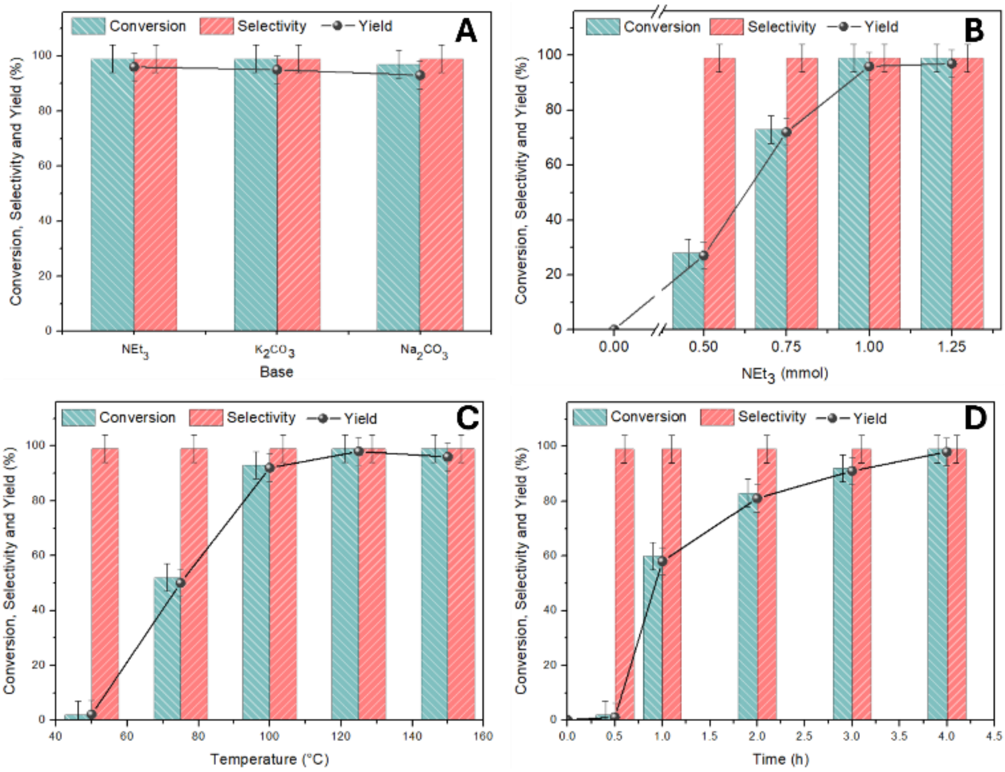

Figure S12: **A**: Study of the influence of the employed base in Heck-Mizoroki reaction. Iodobenzene (1 mmol), ethyl acrilate (1.5 mmol), base (1.5 mmol), 1Pd/CNi (10 mg), GVL (2 ml), 150 °C, 4 h. **B:** Study of the influence of the base concentration in Heck-Mizoroki reaction. Iodobenzene (1 mmol), ethyl acrylate (1.5 mmol), 1Pd/CNi (10 mg), GVL (2 ml), 150 °C, 4 h. **C:** Temperature optimization of Heck-Mizoroki reaction. Iodobenzene (1 mmol), ethyl acrilate (1.5 mmol), NEt_3 (1 mmol), 1Pd/CNi (10 mg), GVL (2 ml), 4 h. **D:** Time optimization of Heck reaction. Iodobenzene (1 mmol), ethyl acrilate (1.5 mmol), NEt_3 (1 mmol), 1Pd/CNi (10 mg), GVL (2 ml), 125 °C.

Table S6. Substrate scope for the Heck-Mizoroki reaction.

Entry	Aryl halide	Alkene	Cross-coupling Product	Conversion [%]
1	Iodobenzene	Ethyl acrylate	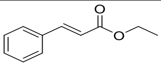	99
2	4-Iodoacetophenone	Ethyl acrylate	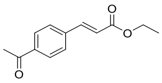	72
3	4-Iodoaniline	Ethyl acrylate	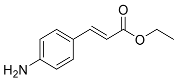	62
4	4-Iodoanisole	Ethyl acrylate	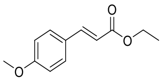	76
5	3-Iodobenzonitrile	Ethyl acrylate	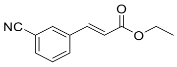	74
6	Bromobenzene	Ethyl acrylate	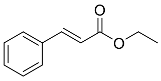	/
7	4-Bromotoluene	Ethyl acrylate	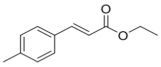	/
8	3-Bromotoluene	Ethyl acrylate	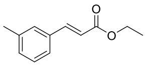	/
9*	4-cloro-1-iodobenzene	Ethyl acrylate	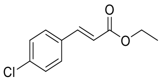	/
10	4-Chlorotoluene	Ethyl acrylate	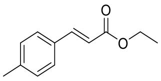	/
11	4-Cl-nitrobenzene	Ethyl acrylate	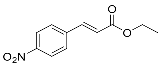	/
12	Iodobenzene	Methyl acrylate	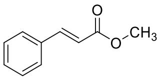	64
13	4-iodoanisole	Methyl acrylate	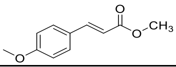	80

Reaction conditions: aromatic halide (1 mmol), alkene (1.5 mmol), NEt_3 (1 mmol), 1Pd/CNi (10 mg), 125 °C, 4 h.

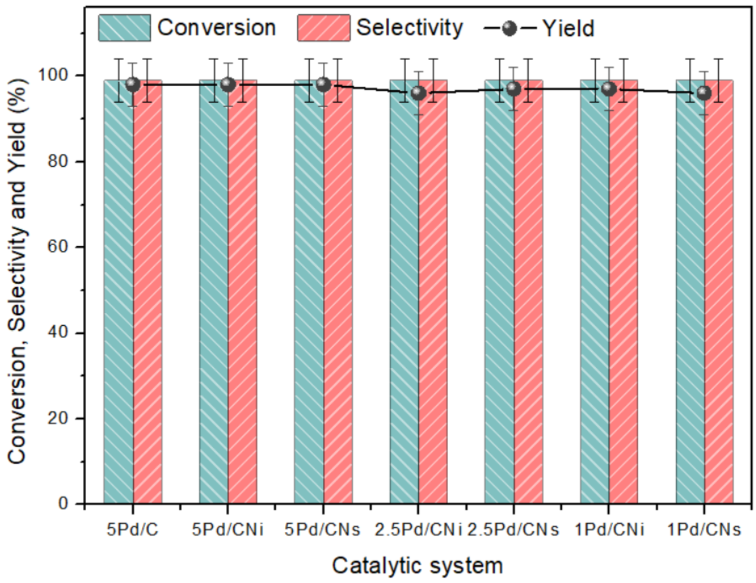

Figure S13: Catalyst screening of Suzuki-Miyaura cross-coupling reaction. Iodobenzene (0.25 mmol), phenylboronic acid (1.5 eq.), K_2CO_3 (2 eq.), catalyst (10 mg), EtOH (3 ml), 75 °C, 4 h.

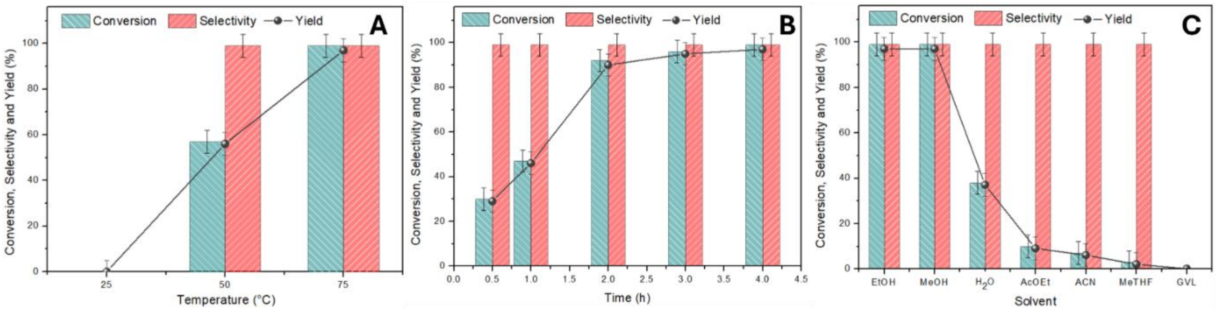

Figure S14. A: Temperature optimization of Suzuki-Miyaura cross-coupling reaction. Iodobenzene (0.25 mmol), phenylboronic acid (1.5 eq.), K_2CO_3 (2 eq.), 1Pd/CNi (10 mg), EtOH (3 ml), 4 h. **B**: Time optimization of Suzuki-Miyaura cross-coupling reaction. Iodobenzene (0.25 mmol), phenylboronic acid (1.5 eq.), K_2CO_3 (2 eq.), 1Pd/CNi (10 mg), EtOH (3 ml), 75 °C. **C**: Solvent screening of Suzuki-Miyaura cross-coupling reaction. Iodobenzene (0.25 mmol), phenylboronic acid (1.5 eq.), K_2CO_3 (2 eq.), 1Pd/CNi (10 mg), solvent (3 ml), 75 °C, 4 h.

Table S7. Substrate scope for the Suzuki-Miyaura cross-coupling reaction.

Entry	Aryl halide	Arylboronic acid	Cross-coupling Product	Conversion [%]	Selectivity [%]
1	iodobenzene	Phenylboronic acid	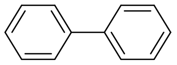	99	99
2	iodobenzene	*p*-tolilboronic acid	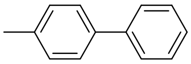	99	95
3	iodobenzene	*m*-tolilboronic acid	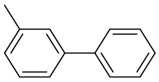	91	97
4	iodobenzene	4-metoxyphenylboronic acid	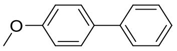	95	96
5	4-iodoaniline	Phenylboronic acid	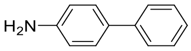	18	99
6	4-iodoanisole	Phenylboronic acid	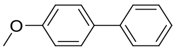	47	99
7	4-I-acetophenone	Phenylboronic acid	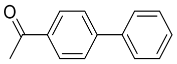	99	99
8	3-I-benzonitrile	Phenylboronic acid	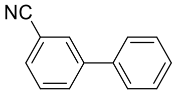	96	99
9	Bromobenzene	Phenylboronic acid	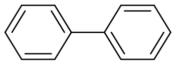	6	99
10	4-Bromotoluene	Phenylboronic acid	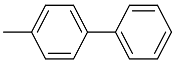	4	99
11	4-Chlorotoluene	Phenylboronic acid	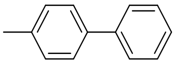	0	0
12	4-Cl-nitrobenzene	Phenylboronic acid	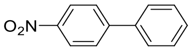	3	99

Reaction conditions: aryl halide (0.25 mmol), arylboronic acid (1.5 eq.), K_2CO_3 (0.5 eq.), **1Pd/CNi** (10 mg), ethanol (3 mL), 75 °C, 4 h.

4. Catalyst recyclability

XRD analysis revealed that both the fresh Pd/CNi and R-1Pd/CNi materials exhibited similar patterns. In both cases, a signal at around 24.0° was observed, indicating the presence of amorphous carbon, especially on the (002) crystallographic plane associated with stacked graphene-like sheets. Additionally, both samples displayed signals at 39.9° and 46.5° (broad, but still discernible from noise), corresponding to the (111) and (200) planes of Pd(0) with a face-centred cubic crystal structure. However, as shown in Figure S15, the XRD pattern of the R-1Pd/CNi catalytic system exhibits lower signal intensities compared to the fresh sample. This could be attributed to certain Pd leaching under the reaction conditions used in the Heck reaction, or to the adsorption of organic moieties, that overlap with the Pd signals. Another possible hypothesis is a structural reorganization during the reaction that leads to an increase in the amorphous component (with a loss of crystallinity) of the catalyst. Nevertheless, it is noteworthy that these phenomena have a minor effect, considering the excellent catalytic activity of the material, which maintains high conversions.

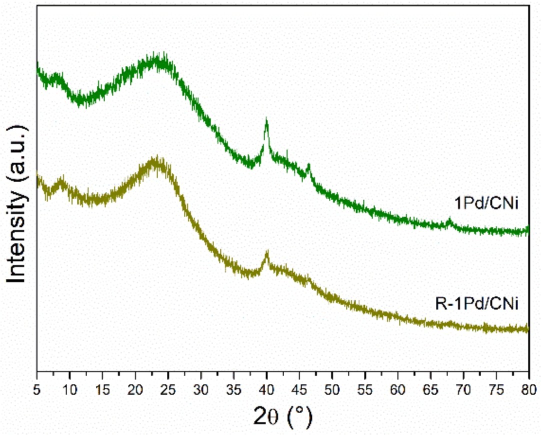

Figure S15: XRD patterns of catalytic systems **1Pd/CNi** and **R-1Pd/CNi.**

The morphology of the catalytic materials, containing 1% Pd loading, was analysed using TEM, and the resulting micrographs are presented in Figure S16. Both 1Pd/CNi and R-1Pd/CNi exhibited highly uniform and well-dispersed palladium nanoparticles supported on a lamellar *N*-doped carbon matrix. As shown, the fresh and spent catalysts did not show significant differences in morphology, which is consistent with constant catalytic activity. The average particle size was determined to be (12.1 ± 1) nm and (13.6 ± 1) nm for the 1Pd/CNi and R-1Pd/CNi materials, respectively. The TEM-EDX images in Figure S17 further reveals, in addition to the Pd nanoparticles, the presence of C, O, and N, all uniformly dispersed within the sample.

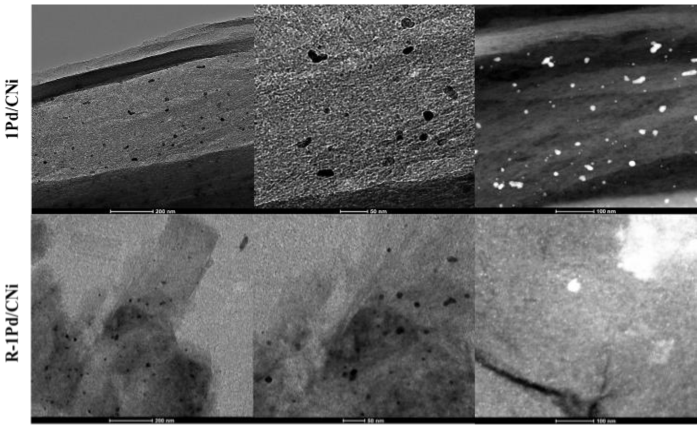

Figure S16. TEM micrograph of 1Pd/CNi and R-1Pd/CNi.

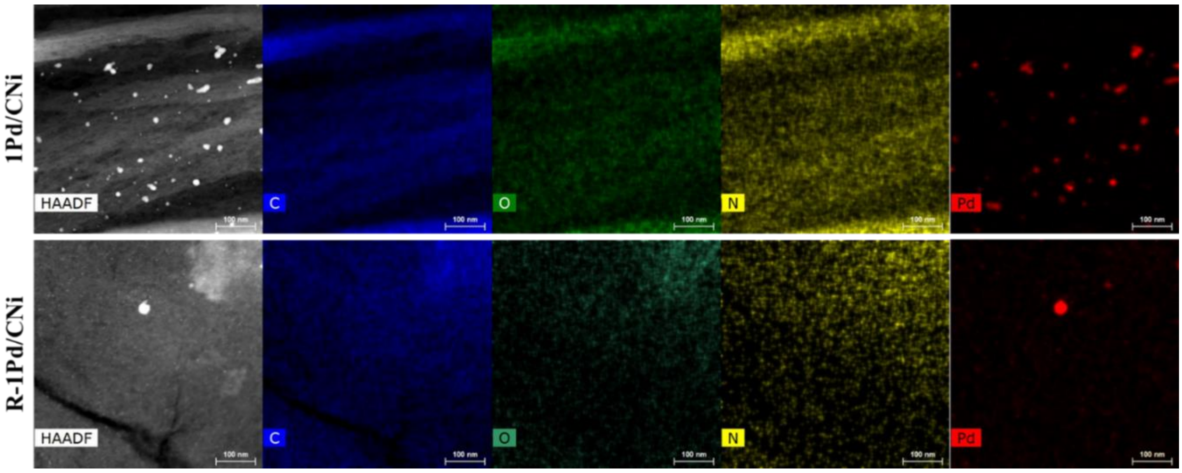

Figure S17. TEM-EDX images of C, O, N, Pd of 1Pd/CNi and R-1Pd/CNi.

The textural properties of the samples were investigated using N_2-physisorption measurements (Table S8). As depicted in Figure S18, the shape of the isotherm remained consistent, yielding a Type IV with a Type II hysteresis loop, indicating a mesoporous behaviour. However, in the recycled material, a reduction in the total pore volume (0.38 cm^3/g instead of 0.48 cm^3/g) and consequently a decrease in surface area (395 m^2/g instead of 526 m^2/g) were observed. This observation could more strongly support, compared to other hypotheses, the idea that after the reaction there is an adsorption of organic molecules on the catalyst, resulting in the occlusion of smaller pores during the reaction. This is also corroborated by the slight increase in the mean pore size (3.8 nm instead of 3.6 nm).

Table S8: Textural properties obtained by N_2-physisorption of 1Pd/CNi and R-1Pd/CNi.

Material	S_{BET} (m^2/g)	D_{BJH} (nm)	V_{BJH} (cm^3/g)
**1Pd/CNi**	526	3.6	0.48
**R-1Pd/CNi**	395	3.8	0.38

** S_{BET}: specific surface area was calculated using the Brunauer-Emmett-Teller (BET) equation. D_{BJH}: mean pore size diameter was calculated using the Barret-Joyner-Halenda (BJH) equation. V_{BJH}: pore volumes were calculated using the Barret-Joyner-Halenda (BJH) equation.*

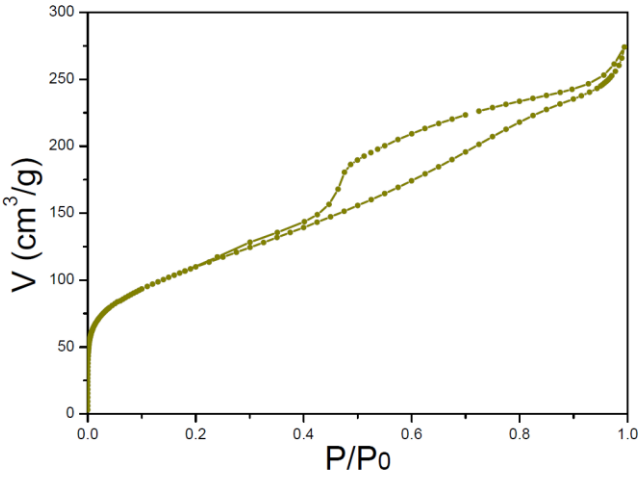

Figure S18: N_2 physisorption isotherm of **R-1Pd/CNi** catalytic system**.**

The chemical composition and nature on the catalysts surface were examined through XPS analyses. The spectra obtained for the R-1Pd/CNi material, as displayed in Figure S19, are similar to those of the corresponding 1Pd/CNi catalytic system; therefore, similar conclusions can be drawn. An apparent difference can be observed in the analysis of the Pd 3*d* region of the XPS spectra (as shown in Figure S19) where the deconvolution of the signal into four components, located at (335.1 ±0.2) eV, (336.2±0.2) eV, (340.3±0.2) eV, and (341.5±0.2) eV, results in more intense signals related to Pd(II). While in the 1Pd/CNi catalytic system, the Pd(0): Pd(II) ratio on the surface is approximately 3:1, in the recycled catalytic system, this ratio decreases to approximately 1:1, indicating a partial oxidation of the catalyst during the reaction. Taking into account the results of XRD, it can be concluded that the catalyst, when considering both the core and the surface, maintains a general prevalence of metallic Pd.

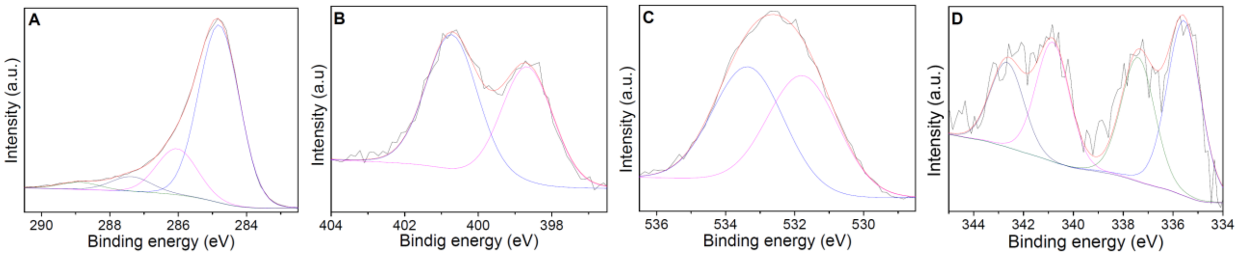

Figure S19. High resolution XPS spectra of **R-1Pd/CNi.** In the (A) C 1s, (B) N1s, (C) O 1s and (D) Pd 3d XPS regions.

5. Characterization analysis of mechanochemically prepared materials

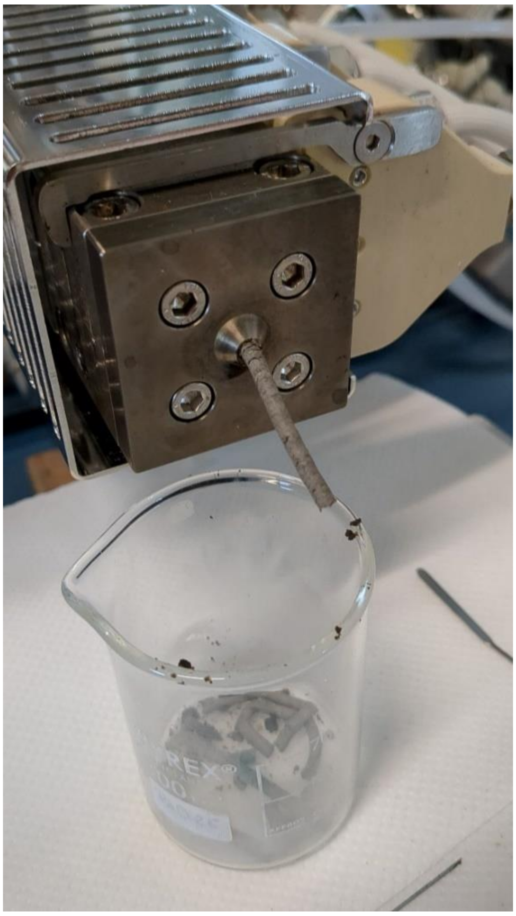

Figure S20: Resulting catalytic material after the extrusion process.

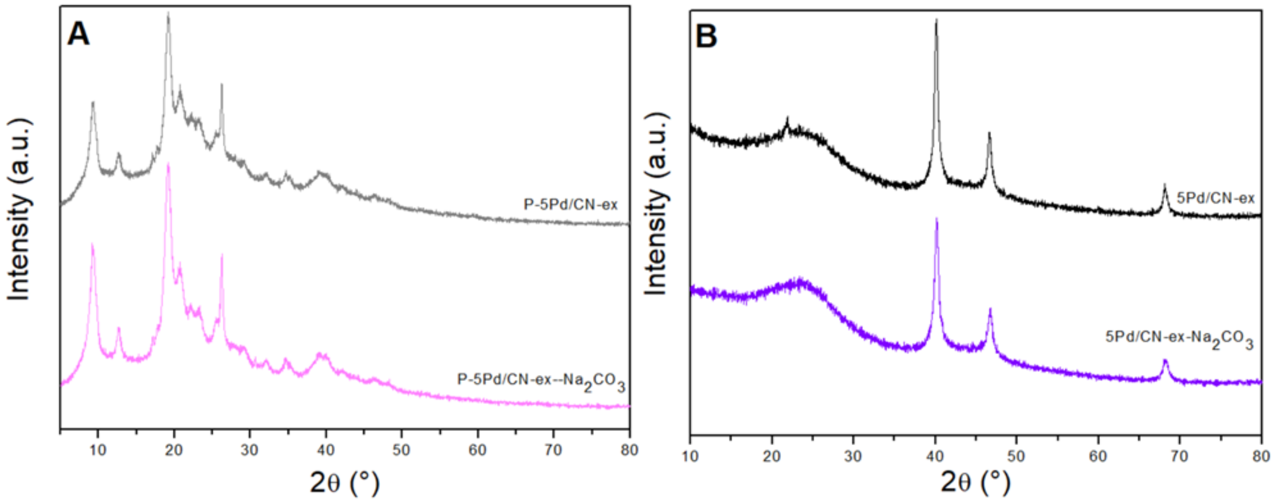

Figure S21: XRD patterns of the catalytic systems before (A) and after (B) thermal treatment.

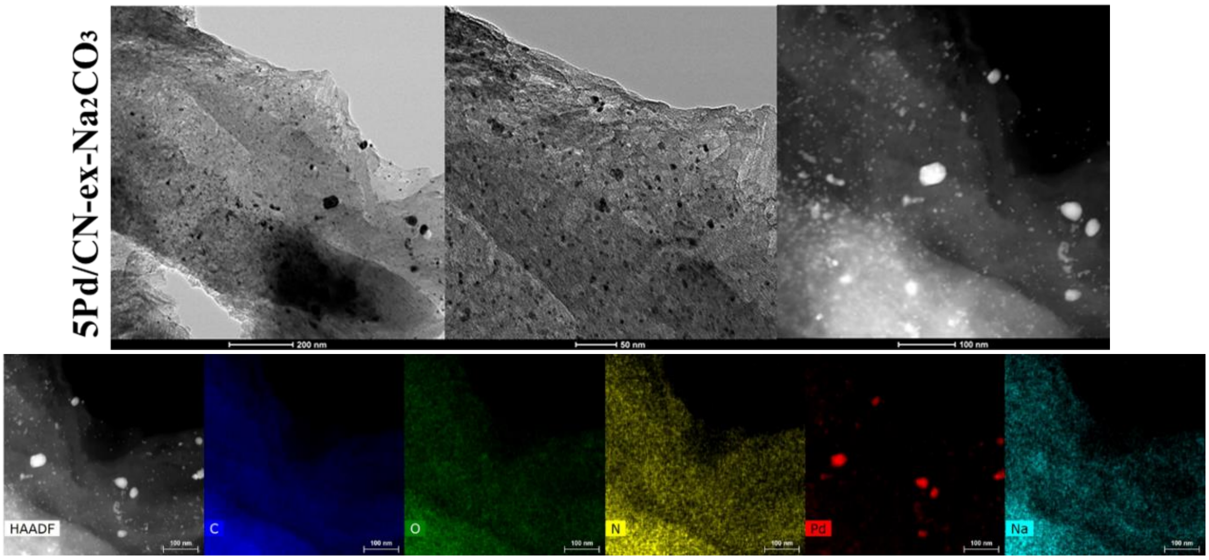

Figure S22: HRTEM, STEM and EDX-mapping micrographs of **5Pd/CN-ex-Na2CO3**.

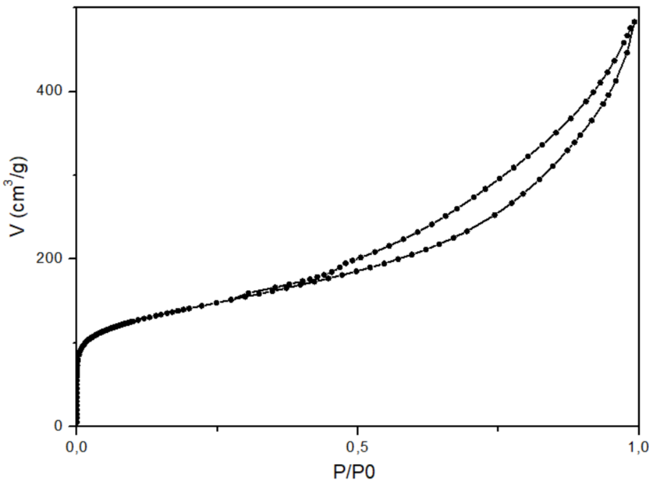

Figure S23: Representative N2 physisorption isotherm of **5Pd/CN-ex**.

Table S9: Textural properties, particle size, and palladium content of the catalytic materials.

Material	S_{BET} (m²/g)	D_{BJH} (nm)	V_{BJH} (cm³/g)	Pd wt.% (ICP-OES)	Pd Particle Size (nm)
**5Pd/CN-ex**	498	4.9	0.61	4.3	14.9
**5Pd/CN-ex-Na_2CO_3**	452	5.3	0.53	3.9	10.3

* S_{BET}: specific surface area was calculated using the Brunauer-Emmett-Teller (BET) equation. D_{BJH}: mean pore size diameter was calculated using the Barret-Joyner-Halenda (BJH) equation. V_{BJH}: pore volumes were calculated using the Barret-Joyner-Halenda (BJH) equation. Pd concentrations were determined by ICP-OES and e Pd particle size was calculated using XRD analyses employing the Scherrer Equation.

Complete XPS analysis of **5Pd/CN-ex** and **5Pd/CN-ex-Na2CO3**. The spectra generally exhibit similar characteristics to those of materials obtained through the more conventional methods A and B. However, for the samples prepared using Na_2CO_3, the surface of the catalytic material displays the presence of not only carbon, oxygen, nitrogen, and palladium but also sodium. In particular, the latter exhibited a distinct Na 1*s* signal at (1071.4 ± 0.2) eV, indicating the presence of Na^+, most likely in the form of Na_2CO_3 (Figure S24I). C 1*s* core level spectra (Figure S24A and S24B) were deconvoluted into four contributions, located at (284.3 ± 0.2) eV, (285.7 ± 0.2) eV, (287.2 ± 0.2) eV and (288.6 ± 0.2) eV, attributed to C–C/C=C bonds from graphitic and/or aromatic carbon, C–OH, C–N/C–O and C=O species, respectively. Similarly to the samples obtained through methods A and B, N 1*s* core level spectra (Figure S24B and S24F) showed the presence of two main bands at (398.4 ± 0.2) eV and (400.3 ± 0.2) eV, which are typically attributed to pyridinic and pyrrolic nitrogen species, respectively. Furthermore, the O 1*s* core level spectra were deconvoluted into two contributions located at (531.2 ± 0.2) eV and (533.2 ± 0.2) eV, which are assigned to lattice O-Metal bonds in metal oxides and to the presence of adsorbed H_2O in the catalysts structure, respectively. This behaviour aligns with that observed in the other synthesized samples. More interesting, an assessment of the chemical composition of the palladium entities on the catalysts surface, specifically for 5Pd/CN-ex and 5Pd/CN-ex-Na_2CO_3, was conducted based on the Pd 3*d* core level spectra. Notably, these spectra displayed marked differences from all other samples (refer to Figure S24D and S24H). In this case, the Pd3*d* signals were deconvoluted into six distinct contributions. Four of these contributions correspond to the previously mentioned ones, located at (335.1 ± 0.2) eV and (340.3 ± 0.2) eV referred respectively to the doublet Pd $3d_{5/2}$ – Pd $3d_{3/2}$ of Pd(0), and at (336.2 ± 0.2) eV and (341.5 ± 0.2) eV associated respectively to the doublet Pd$3d_{5/2}$ Pd$3d_{3/2}$ of Pd(II) oxide. In addition to these signals, two additional contributions can be observed, located at (343.6 ± 0.2) eV and (338.3 ± 0.2) eV, indicating the presence of Pd-*N* bonds.

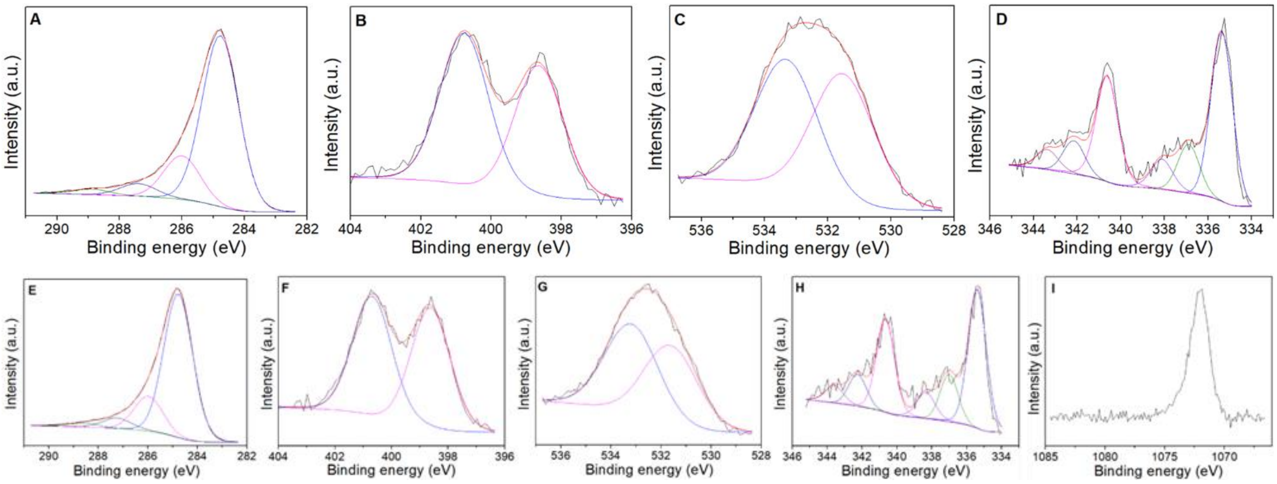

Figure S24: High resolution XPS spectra of **5Pd/CN-ex** (A-D) and **5Pd/CN-ex-Na2CO3** (E-I). For each catalytic system, it's represented in this order the C 1*s*, N 1*s*, O 1s and Pd 3*d* XPS regions. For **5Pd/CN-ex-Na2CO3** it's also available the Na 1s region.

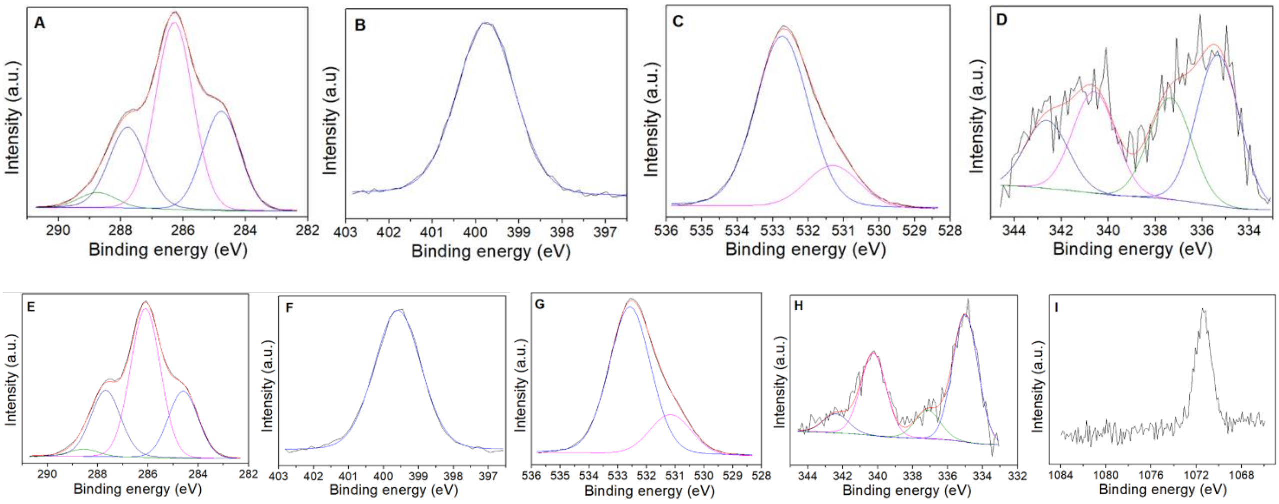

Figure S25: XPS spectra of **P-5Pd/CN-ex** (A-D) and **P-5Pd/CN-ex-Na_2CO_3** (E-I). For each catalytic system, it's represented in this order the C 1*s*, N 1*s*, O 1*s* and Pd 3*d* XPS regions. For **P-5Pd/CN-ex-Na_2CO_3** it's also available the Na1*s* region.

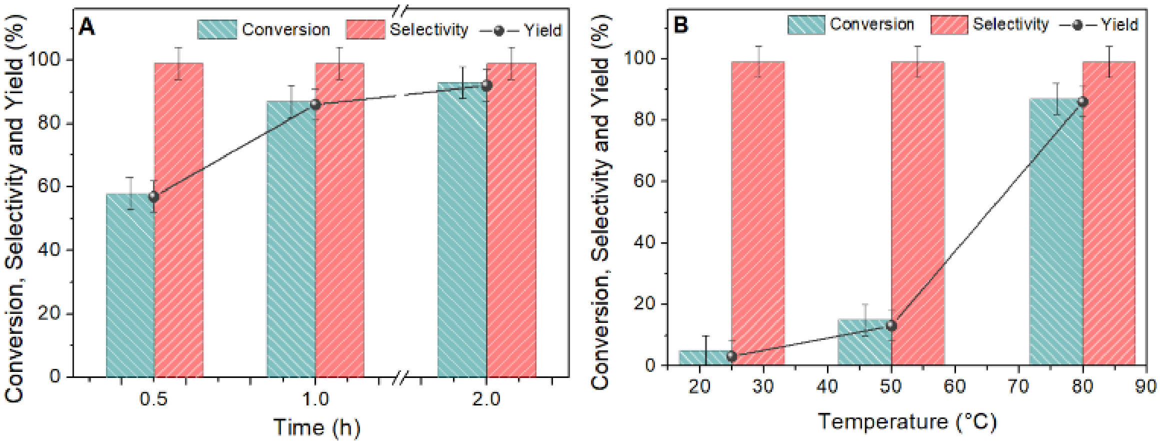

Figure S26. (A) Time and (B) Temperature optimization. Iodobenzene (4 mmol), phenylboronic acid (1.5 eq.), K_2CO_3 (2 eq.), 1Pd/CNi (40 mg), 50 rpm of velocity of twin screw.

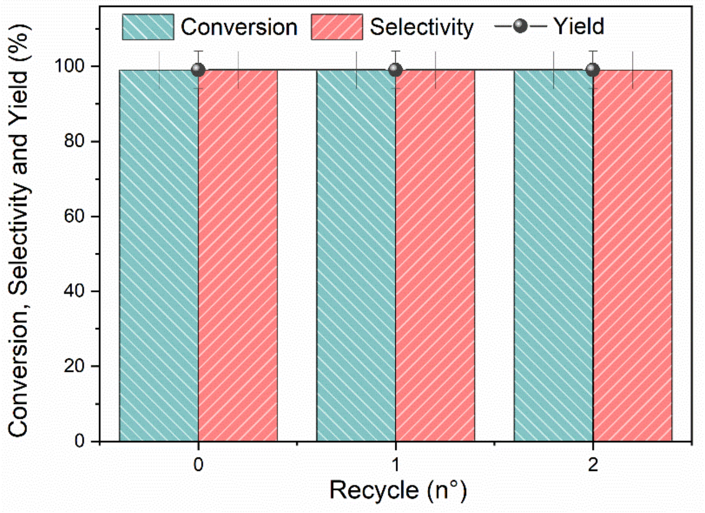

Figure S27: Recyclability study of Heck-Mizoroki cross-coupling reaction. 5Pd/CN-ext (10 mg), iodobenzene (1 mmol), ethyl acrylate (1.5 mmol), NEt3 (1.5 mmol), GVL (2 ml), 125 °C, 4 h.

[15] D. Polidoro, A. Perosa, E. Rodríguez-Castellón, P. Canton, L. Castoldi, D. Rodríguez-Padrón, M. Selva, *ACS Sustain Chem Eng* **2022**, *10*, 13835–13848.

[55] Y. Huang, H. Yan, C. Zhang, Y. Wang, Q. Wei, R. Zhang, *Nanomaterials* **2021**, *11*, 2776.

[58] D. Polidoro, D. Rodriguez-Padron, A. Perosa, R. Luque, M. Selva, *Materials* **2023**, *16*, DOI 10.3390/ma16020575.

[67] Q. Wu, L. Wang, B. Zhao, L. Huang, S. Yu, A. J. Ragauskas, *J Colloid Interface Sci* **2022**, *605*, 82–90.

[68] C. Xu, A. R. Puente-Santiago, D. Rodríguez-Padrón, A. Caballero, A. M. Balu, A. A. Romero, M. J. Muñoz-Batista, R. Luque, *ACS Appl Energy Mater* **2019**, *2*, 2161–2168.

[69] D. Polidoro, A. Perosa, M. Selva, D. Rodríguez-Padrón, *ChemCatChem* **2023**, *15*, DOI 10.1002/cctc.202300415.

[70] R. Kumar, J. H. Oh, H. J. Kim, J. H. Jung, C. H. Jung, W. G. Hong, H. J. Kim, J. Y. Park, I. K. Oh, *ACS Nano* **2015**, *9*, 7343–7351.

[71] D. Rigo, D. Polidoro, L. Marcuzzo, A. Perosa, M. Selva, *ACS Sustain Chem Eng* **2023**, *11*, 12602–12613.
